# Inhibition of mPGES-1 or COX-2 Results in Different Proteomic and Lipidomic Profiles in A549 Lung Cancer Cells

**DOI:** 10.3389/fphar.2019.00636

**Published:** 2019-06-07

**Authors:** Filip Bergqvist, Elena Ossipova, Helena Idborg, Joan Raouf, Antonio Checa, Karin Englund, Petter Englund, Payam Emami Khoonsari, Kim Kultima, Craig E. Wheelock, Karin Larsson, Marina Korotkova, Per-Johan Jakobsson

**Affiliations:** ^1^Rheumatology Unit, Department of Medicine, Solna, Karolinska Institutet, Karolinska University Hospital, SE-171 76 Stockholm, Sweden; ^2^Division of Physiological Chemistry 2, Department of Medical Biochemistry and Biophysics, Karolinska Institutet, Stockholm, Sweden; ^3^Department of Analytical Chemistry, Stockholm University, Stockholm, Sweden; ^4^Department of Medical Sciences, Clinical Chemistry, Uppsala University, Uppsala, Sweden

**Keywords:** microsomal prostaglandin E synthase-1 inhibitor, prostaglandin E_2_, cancer, inflammation, cyclooxygenase-2 inhibitor

## Abstract

Pharmacological inhibition of microsomal prostaglandin E synthase (mPGES)-1 for selective reduction in prostaglandin E_2_ (PGE_2_) biosynthesis is protective in experimental models of cancer and inflammation. Targeting mPGES-1 is envisioned as a safer alternative to traditional non-steroidal anti-inflammatory drugs (NSAIDs). Herein, we compared the effects of mPGES-1 inhibitor Compound III (CIII) with the cyclooxygenase (COX)-2 inhibitor NS-398 on protein and lipid profiles in interleukin (IL)-1β-induced A549 lung cancer cells using mass spectrometry. Inhibition of mPGES-1 decreased PGE_2_ production and increased PGF_2α_ and thromboxane B_2_ (TXB_2_) formation, while inhibition of COX-2 decreased the production of all three prostanoids. Our proteomics results revealed that CIII downregulated multiple canonical pathways including eIF2, eIF4/P70S6K, and mTOR signaling, compared to NS-398 that activated these pathways. Moreover, pathway analysis predicted that CIII increased cell death of cancer cells (*Z* = 3.8, *p* = 5.1E−41) while NS-398 decreased the same function (*Z* = −5.0, *p* = 6.5E−35). In our lipidomics analyses, we found alterations in nine phospholipids between the two inhibitors, with a stronger alteration in the lysophospholipid (LPC) profile with NS-398 compared to CIII. Inhibition of mPGES-1 increased the concentration of sphinganine and dihydroceramide (C_16:0_DhCer), while inhibition of COX-2 caused a general decrease in most ceramides, again suggesting different effects on cell death between the two inhibitors. We showed that CIII decreased proliferation and potentiated the cytotoxic effect of the cytostatic drugs cisplatin, etoposide, and vincristine when investigated in a live cell imaging system. Our results demonstrate differences in protein and lipid profiles after inhibition of mPGES-1 or COX-2 with important implications on the therapeutic potential of mPGES-1 inhibitors as adjuvant treatment in cancer. We encourage further investigations to illuminate the clinical benefit of mPGES-1 inhibitors in cancer.

## Introduction

Prostaglandin E_2_ (PGE_2_) is a key regulatory lipid mediator in inflammation, immune responses, and tumor development (Wang and Dubois, 2010; Ricciotti and FitzGerald, 2011; Kalinski, 2012; Nakanishi and Rosenberg, 2013). It is a metabolite of arachidonic acid, which is released from membrane phospholipids by cytosolic phospholipase A2 (cPLA_2_) and then converted by cyclooxygenase (COX)-1/2 into unstable PGH_2_ that is further processed by terminal synthases into the major prostanoids including PGE_2_, PGD_2_, PGF_2α_, PGI_2_, and thromboxane A_2_ (TXA_2_) (Smith et al., 2011). Various non-steroidal anti-inflammatory drugs (NSAIDs), which frequently are used to treat inflammation and pain, reduce the level of PGE_2_ and are able to promote anti-proliferative effects in different cancer models (Wang and Dubois, 2010). Despite the promising potential for cancer treatment, NSAIDs are associated with severe adverse effects (Sorensen et al., 2000; Cheng et al., 2006; Grosser et al., 2006). Hence, our focus has now shifted downstream of cyclooxygenases to the development of microsomal prostaglandin E synthase-1 (mPGES-1) inhibitors to selectively reduce PGE_2_ biosynthesis and spare the other prostanoids (Larsson and Jakobsson, 2015). Genetic deletion as well as pharmacological inhibition of mPGES-1 have demonstrated this target as an effective anti-cancer regimen (Hanaka et al., 2009; Kamei et al., 2009; Nakanishi et al., 2011; Sasaki et al., 2012; Howe et al., 2013; Takahashi et al., 2014; Zelenay et al., 2015; Kock et al., 2018).

**Figure 1:**
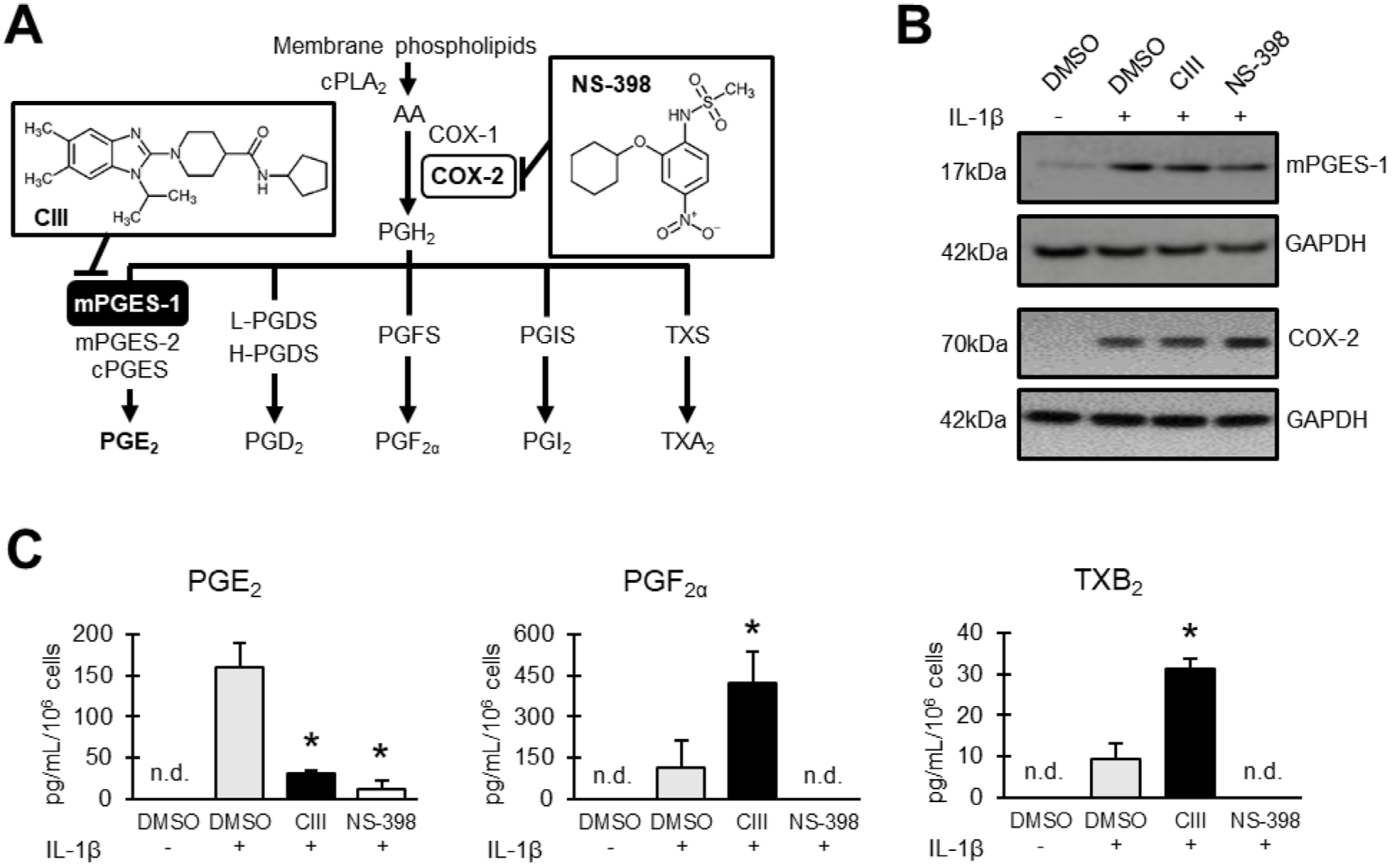
Effect on prostanoid production by CIII and NS-398. **(A)** Schematic overview of prostanoid biosynthesis. Arachidonic acid (AA) is released from phospholipids in cell membranes by action of cPLA_2_ and then converted into PGH_2_ *via* COX-1/2 enzymes. The key terminal enzyme for PGE_2_ production during inflammatory conditions is mPGES-1. Inserted molecules are mPGES-1 inhibitor CIII and COX-2 inhibitor NS-398. **(B)** Representative Western blot showing induced expression of mPGES-1 and COX-2 with IL-1β in A549 cells. **(C)** Effect on prostanoid production as quantified in A549 cell supernatant with LC-MS/MS. Results are presented as mean ± SD (*n* = 3) for one experiment. Statistical significance was tested using ANOVA followed by individual *t* test with Bonferroni correction (*p* < 0.05). The asterisk (*) represents significance to IL-1β. n.d = not detected.

Non-small cell lung cancer (NSCLC) is the most common and deadliest lung cancer. One third of NSCLC tumors have a mutation in the *KRAS* gene, leading to overexpression of MYC and sustained proliferation (Cancer Genome Atlas Research, 2014). Increased MYC activity in lung tumors correlates with increased interleukin (IL)-1β production, higher cPLA_2_ activity, and increased expression of downstream enzyme COX-2, which subsequently results in higher levels of liberated arachidonic acid and eicosanoid production (Hall et al., 2016). IL-1β promotes prostanoid synthesis by activating cPLA_2_ translocation and inducing COX-2 expression *via* several mechanisms (Harper and Tyson-Capper, 2008) including sphingolipid metabolism (Billich et al., 2005). This creates a vicious cycle with elevated activity of the COX-2/mPGES-1/PGE_2_ pathway in lung tumors. Indeed, COX-2 and mPGES-1 are found overexpressed in NSCLC (Yoshimatsu et al., 2001; Petkova et al., 2004) and overexpression of either enzyme correlates with poor prognosis (Khuri et al., 2001; Wu et al., 2010). However, mPGES-1 overexpression and increased PGE_2_ production alone is not sufficient to promote lung cancer *in vivo* (Blaine et al., 2005). The expressions of mPGES-1 and COX-2 are induced upon treatment with IL-1β in the NSCLC cell line A549 (Jakobsson et al., 1999), and this system is appreciated as a good model to study the modulation of the COX-2/mPGES-1/PGE_2_ pathway. We have shown that knockdown of mPGES-1 in A549 cells delays tumor growth in a mouse xenograft model (Hanaka et al., 2009). In addition, our group has characterized a selective mPGES-1 inhibitor, Compound III (CIII, **Figure 1A**), which has activity in both human and rodent systems (Leclerc et al., 2013; Ozen et al., 2017; Kock et al., 2018; Nishizawa et al., 2018). We recently reported that daily treatment with CIII reduced tumor progression in two pre-clinical models of neuroblastoma (Kock et al., 2018).

While many of the studies conducted in the field of COX-1/2 and mPGES-1 inhibitors are specifically focusing on the eicosanoid profile, there is a lack of knowledge regarding the effects on the proteome and lipidome when targeting key enzymes in the biosynthesis of PGE_2_. In this study, we aimed to examine the effects on proteins and lipids in IL-1β-induced A549 cells after pharmacological inhibition of mPGES-1 or COX-2.

## Materials and Methods

### Cell Culture

A549 cells (ATCC, Manassas, VA, USA) were cultured in Dulbecco’s modified eagle medium (DMEM), supplemented with 10% fetal bovine serum (FBS), 100 U/ml penicillin, 100 μg/ml streptomycin, 1 mM sodium pyruvate, and 6 mM glutamine (all purchased from Sigma-Aldrich) at 37°C in a humidified atmosphere containing 5% CO_2_. Cells were seeded in 175-cm^2^ flasks with ventilated cap at a density of 2 million cells/flask and grown to 85% cell confluency. At confluency, the cells were induced with 5 ng/ml of IL-1β (R&D Systems) in fresh medium and treated with either 10 μM mPGES-1 inhibitor Compound III (CIII) (NovaSAID AB, Sweden) or 0.1 μM COX-2 inhibitor NS-398 (Sigma-Aldrich), previously tested to yield reduction in PGE_2_ biosynthesis to the same degree during these settings (Leclerc et al., 2013). Untreated cells were supplemented with 0.05% v/v of dimethyl sulfoxide (DMSO, Sigma-Aldrich) as vehicle control. This resulted in four experimental conditions, referred to as *Ctrl* (DMSO), *IL-1β* (IL-1β + DMSO), *CIII* (IL-1β + CIII), and *NS-398* (IL-1β + NS-398). After 24-h treatment, cell supernatants were collected and immediately stored at −80°C for prostanoid profiling. The cells were washed once with PBS (Sigma-Aldrich) and detached using trypsin–EDTA (Sigma-Aldrich). Viable cells were counted by staining with trypan blue (Sigma-Aldrich). The cells were pelleted at 300 × *g* for 5 min and then stored at −80°C until further processed. All experimental conditions were performed in technical triplicates. The cell culture experiments were performed in separate batches for proteomics and lipidomics analyses. Each batch of experiments, i.e., four conditions in triplicates, were quality controlled by staining of mPGES-1 and COX-2 using Western blot and by measuring extracellular PGE_2_ using prostanoid profiling as described below. Cell cultures were routinely checked for mycoplasma contamination using MycoAlert^™^ PLUS kit (Lonza, Switzerland) according to the manufacturer’s instructions.

### Western Blot

A549 cell pellets were lysed in Tissue Protein Extraction Reagent (T-PER, Thermo Fisher Scientific) supplemented with 1× complete protease inhibitor cocktail (Roche Diagnostics) on ice for 30 min. The total protein concentration was determined by measuring absorbance at 260 nm with NanoDrop (Thermo Fischer Scientific). Forty micrograms of protein was separated by gel electrophoresis on NuPage^®^ Novex^®^ Bis–Tris gel system (Invitrogen AB, Sweden). Proteins were transferred to a polyvinylidene difluoride (PVDF) membrane by using a Trans-Blot SD semi-dry transfer cell (Bio-Rad Laboratories AB, Sweden), and the membrane was blocked with 5% milk (Bio-Rad Laboratories AB, Sweden) in PBS containing 0.1% Tween 20 (Sigma-Aldrich) for 30 min on a shaker at RT. Subsequently, the membrane was incubated with polyclonal antibody against either mPGES-1 [in-house (Westman et al., 2004)] or COX-2 (Cayman Chemicals, USA) at 4°C overnight. Secondary antibody horseradish-peroxidase-coupled anti-rabbit IgG from donkey (GE Healthcare, Sweden) was incubated with membrane for 1 h at RT. The membrane was then washed 3 × 10 min in PBS containing 0.1% Tween 20, and protein bands were visualized by using Enhanced Chemiluminescence kit (GE Healthcare, Sweden) on an Amersham Hybond film (GE Healthcare, UK). GAPDH antibody (Invitrogen AB, Sweden) was used as a protein loading control.

### Proteomics

#### Sample Preparation for Proteomics

Microsomal and soluble protein fractions of A549 cell pellets were prepared as described elsewhere (Eriksson et al., 2008). The microsomes were solubilized in 50 mM TEAB buffer and delipidated using MeOH/CHCl_3_ precipitation according to Wessel and Flügge (1984). Protein pellets were solubilized in 50 mM TEAB containing 0.01% SDS and 0.1% (w/v) RapiGest SF Surfactant (Waters). The protein concentration was determined using the Pierce^™^ BCA Protein Assay kit (Thermo Fisher Scientific). Equal aliquots of total protein obtained for soluble and microsomal fractions were subsequently reduced using 200 mM DTT for 30 min at 56°C and then alkylated using 1 M iodoacetamide for 1 h at RT in the dark. Two-step proteolytic digestion was performed, starting with digestion using Lys-C (1:50, w/w) for 2 h at 37°C followed by digestion with trypsin (1:100, w/w) overnight at 37°C. Samples were acidified using 100% formic acid, and RapiGest SF Surfactant was removed by centrifugation. Supernatants were loaded onto Strata-X solid-phase extraction (SPE) columns (Phenomenex) and peptides were eluted with 80% MeCN in 0.2% formic acid and evaporated to dryness under vacuum. Stable isotope dimethyl labeling of obtained peptides utilizing light and intermediate labels was performed according to Boersema et al. (2009). Briefly, dried peptides were suspended in one volume of 100 mM TEAB followed by addition of 0.3% (v/v) of CH_2_O (Sigma-Aldrich) to Ctrl and 0.3% (v/v) of CD_2_O (Sigma-Aldrich) to IL-1β, CIII, and NS-398. One volume of 0.6 M NaBH_3_CN was added to all samples that were incubated for 1 h at RT and then the reaction was quenched by adding NH_4_OH and formic acid. Finally, the deuterated samples (IL-1β, CIII, and NS-398) for soluble and membrane fraction were combined with equal amount of Ctrl sample from the corresponding fraction. The samples were then loaded on Strata-X SPE columns and the stable isotope-labeled peptides were eluted with 80% MeCN in 0.2% formic acid and evaporated to dryness under vacuum.

#### High-Performance Liquid Chromatography (HPLC)-Fractionation for Proteomics

In order to reduce sample complexity prior to liquid chromatography tandem mass spectrometry (LC-MS/MS) analysis, off-line peptide fractionation was performed using the Dionex Ultimate 3000 UV HPLC system (Thermo Fisher Scientific). Peptides were loaded in buffer A (20 mM ammonia) on an XBridge peptide BEH300 C18 Column, 300 Å, 3.5 µm, 2.1 × 150 mm (Waters, USA) and separated using a 40-min non-linear gradient of 3–88% B (80% MeCN in 20 mM ammonia) into 12 fractions. Obtained fractions were pooled into four final fractions across the gradient area and evaporated to dryness under vacuum. This resulted in 72 samples (2 cell fractions × 3 treatment groups × 3 replicates × 4 fractions from HPLC-fractionation) for proteomics using LC-MS/MS analysis.

#### Proteomics LC-MS/MS Analysis

LC-MS/MS of peptide fractions was performed on a Dionex Ultimate 3000 HPLC system (Thermo Fisher Scientific) coupled to a Q Exactive Orbitrap mass spectrometer equipped with EASY-Spray ion source (Thermo Fisher Scientific). Peptide samples were trapped on an Acclaim PepMap trap column (C18, 3 µm, 100 Å, 75 µm × 20 mm) and separated on an EASY-Spray PepMap RSLC column (C18, 2 µm, 100 Å, 75 µm × 50 cm, Thermo Fisher Scientific) using a gradient of A (0.1% formic acid) and B (95% MeCN, 0.1% formic acid), ranging from 3% to 40% B in 50 min with a flow of 0.25 µL/min. The top 10 ions were selected for fragmentation, and the survey scan was performed at 70,000 resolution from 300 to 1,600 *m/z*, with a max injection time of 100 ms and a target of 1 × 10^6^ ions. For generation of high energy collision dissociation (HCD) fragmentation spectra, a max ion injection time of 200 ms and AGC of 2 × 10^5^ were used before fragmentation at 30% normalized collision energy, 17,500 resolution. Precursors were isolated with a width of 2 *m/z* and put on the exclusion list for 30 s. Single and unassigned charge states were rejected from precursor selection.

#### Proteomics LC-MS/MS Data Analysis

Obtained tandem mass spectra were converted to open source format (mzML) by “msconvert” from ProteoWizard (Chambers et al., 2012) and processed using OpenMS v2.1.0 (Rost et al., 2016) through the following workflow: The identification was based on the UniProt/Swiss-Pro human database (2016-04) combined with a decoy database. MS-GF+ (Kim and Pevzner, 2014) and Xtandem search engines were used to perform the identification using the following parameters: precursor mass tolerance: 10 ppm; fragment mass tolerance: 0.04 Da (only in Xtandem); charges: 2 to 4; enzyme: trypsin; fixed modifications: Carbamidomethyl (C), Dimethyl, Dimethyl (K and N-term), Dimethyl:2H(4) (K and N-term), Oxidation (M), Deamidated (N and Q); missed cleavages: 2, Instrument: HighRes (only MS-GF+). The unmentioned parameters were set to default value. The search engine scores were converted to posterior error probability using “IDPosteriorErrorProbability” and merged using the “ConsensusID” tool (Nahnsen et al., 2011). *q* values were calculated for peptides using the “FalseDiscoveryRate” tool and peptides with *q* values lower than 0.05 were selected for the next step. For quantification, the raw data were centroided using “PeakPickerHiRes” and features were detected using “FeatureFinderMultiplex” using the following parameters: labels: [Dimethyl0][Dimethyl4]; charge: 2:5, rt_typical: 12 s; rt_min: 3 s; mz_tolerance: 10 ppm; missed_cleavages: 2; intensity_cutoff: 10; peptide_similarity: 0.6; averagine_similarity: 0.5. The identification result was mapped to the quantification result using “IDMapper” allowing 5-s retention time and 5-ppm mass deviation. Finally, the peptides across the samples were linked using “FeatureLinkerUnlabeledQT” allowing 60-s retention time and 10-ppm mass error deviation. Ingenuity Pathway Analysis (Ingenuity Systems, www.ingenuity.com) was applied to identified and quantified protein datasets in order to find altered pathways. The mass spectrometry proteomics data have been deposited to the ProteomeXchange Consortium *via* PRIDE (Perez-Riverol et al., 2019) partner repository with the dataset identifier PXD013490.

### Prostanoid Profiling Using LC-MS/MS

Prostanoids in cell supernatants were spiked with deuterated standards, extracted with SPE, and analyzed using LC-MS/MS according to a previously described method (Idborg et al., 2013). Results are presented in absolute concentration (mean ± SD).

### Lipid Extraction for Fatty Acid Profiling of Total Lipids and Phospholipid Profiling

Lipids were extracted from cell pellets according to a previously described method (Idborg et al., 2013) and dried under a stream of nitrogen. The dried extracts were reconstituted in 500 µl of CHCl_3_/MeOH (1:1), sonicated in a water bath, and then split in two fractions: 150 µl for fatty acid profiling of total lipids using gas chromatography with flame ionization detector (GC-FID) and 350 µl for phospholipid analysis with LC-MS/MS. The fraction for CG-FID analysis was processed directly according to below. Butylated hydroxytoluene (BHT) was added to a final concentration of 0.1 mg/ml in the phospholipids fraction to prevent oxidation of lipids, and the samples were stored at −20°C until analyzed.

#### Fatty Acid Profiling of Total Lipids Using GC-FID

Extracted lipids were derivatized into fatty acid methyl esters (FAMEs) and analyzed with GC-FID according to a previously described method (Idborg et al., 2013). Results are presented in weight% (mean ± SD) of total identified FAMEs within samples.

#### Phospholipid Profiling Using LC-MS/MS

The phospholipid fraction was analyzed using targeted LC-MS/MS according to a previously described method (Raouf et al., 2018). Our targeted method for phospholipids comprised 107 entities, including 11 lysophosphatidylcholines (LPCs), 32 phosphatidylcholines (PCs), 32 phosphatidylethanolamine (PEs), and 32 phosphatidylserine (PSs). Results are presented in relative abundance (area%, mean ± SD) of phospholipid species within phospholipid species classes. Phospholipid species are named based on lipid class (e.g., PC) with total acyl chain length and degree of unsaturation (e.g., 32:2).

### Sphingolipid Profiling Using LC-MS/MS

Sphingolipids are named based on head group (e.g., Cer) with acyl chain length and degree of unsaturation in the amide-linked fatty acid (e.g., C_16:0_). For the analysis of sphingolipids, cell pellets containing around 15 million cells were lysed with 1 ml of MeOH and sonicated in an ultrasound bath for 15 min. Samples were then centrifuged at 10,000 × *g* for 15 min. Two separate methods were used for quantification of sphingolipids.

Extraction method 1: For the analysis of sphingomyelins (SM, *n* = 6), ceramides (Cer, *n* = 9), hexosylceramides (HexCer, *n* = 5), lactosylceramides (LacCer, *n* = 4), and dihydroceramides (DhCer, *n* = 1), an aliquot of 100 µl of the extract was used. First, 10 µl of a mixture containing odd chain sphingolipids (one for each class) was added to the extract. After a vortexing step, 280 µl of MeOH and 190 µl of CHCl_3_ was added to the samples. Extraction was performed by sequential additions of H_2_O, CHCl_3_, and H_2_O (150, 190, and 160 µl, respectively), with a vortexing step after every addition. Samples were then centrifuged at 5,000 × *g* for 10 min and the organic lower layer was transferred to another Eppendorf tube. The organic extract was then evaporated to dryness under vacuum and stored at −80°C until analysis. On the day of analysis, extracts were reconstituted in 200 µl of MeOH, sonicated for 2 min, vortexed, and filtered by centrifugation for 3.5 min at 3,500 × *g* using 0.1-mm membrane spin filters (Merck Millipore, Billerica, MA). Extracts were then transferred into autosampler vials and 7.5 µl was injected for LC-MS/MS analysis.

Extraction method 2: For the analysis of sphingosine, sphinganine, sphingosine-1-phosphate, and sphinganine-1-phosphate, another aliquot of 100 µl of the extract was used. After addition of 10 µl of a mixture containing odd chain sphingoid bases, 100 µl of 0.5% formic acid in water and 200 µl of methanol were added to the samples. Samples were then vortexed and centrifuged at 5,000 × *g* for 10 min and filtered by centrifugation for 3.5 min at 3,500 × *g* using 0.1-mm membrane spin filters. Extracts were then transferred into autosampler vials and 7.5 µl was injected for LC-MS/MS analysis.

Sphingolipids were measured in MRM mode using an Acquity ultra performance liquid chromatography (UPLC) coupled to a Xevo TQ mass spectrometer (Waters, Milford, MA). Two independent chromatographic separations were used on an Acquity UPLC BEH C8 column (1.7 µm, 2.1 mm × 150 mm) equipped with an Acquity UPLC BEH C8 VanGuard precolumn. The chromatographic method and MS/MS selected reaction monitoring transitions are detailed elsewhere (Checa et al., 2015). Results are presented in absolute concentration (mean ± SD).

### Monitoring of Cell Death Using Live Cell Imaging System

Apoptosis assay: A549 cells were seeded at 20,000 cells per well in a 96-well tissue culture plate. The cells were let to attach overnight and treated the next day as specified in the section Cell Culture: *Ctrl* (DMSO), *IL-1β* (IL-1β + DMSO), *CIII* (IL-1β + CIII), and *NS-398* (IL-1β + NS-398). SYTOX Green Nucleic Acid Stain (1:50.000, cat# S7020, Invitrogen AB, Sweden) was added to one set of triplicate wells and Annexin V Red Reagent (1:200, cat# 4641, Essen BioScience) and Caspase-3/7 Green Reagent (1:1,000, cat# 4440, Essen BioScience) were added to another set of triplicate wells. Staurosporine (STS, S5921, Sigma-Aldrich) at 1 µM was used as positive control. The cells were monitored using IncuCyte S3 (Essen BioScience) for 48 h. Four images per well at 10× magnification were collected every third hour. The data were analyzed using IncuCyte S3 version 2018A (Essen BioScience) with individual mask settings for SYTOX Green, Annexin V Red Reagent, and Caspase-3/7 Green Reagent based on green or red fluorescence intensity. Results are presented as green object count per mm^2^ for SYTOX Green and active caspase-3/7 and as red object area (% confluence) for annexin V.

Proliferation assay: A549 cells were seeded at 5,000 cells per well in 96-well tissue culture plates. The cells were let to attach overnight and treated the next day as specified in the section Cell Culture. In addition, cells were treated with cytostatic drugs cisplatin (0.01–100 µM), etoposide (0.01–100 µM), or vincristine (0.001–10 µM) in combination with the treatment stated earlier: *Ctrl* (DMSO), *IL-1β* (IL-1β + DMSO), *CIII* (IL-1β + CIII), and *NS-398* (IL-1β + NS-398). SYTOX Green Nucleic Acid Stain (1:50.000) was used as marker for cell death. The cells were monitored using IncuCyte S3 for 70 h. Four images per well at 10× magnification were collected every third or fourth hour. The data were analyzed using IncuCyte S3 version 2018A with individual mask settings for SYTOX Green (based on green fluorescence intensity) and confluency (phase object). Results are presented as percentage of total well area for confluency and green object count per mm^2^ for SYTOX Green.

### Statistical Analysis

Statistical analyses were performed using GraphPad Prism 6.0 (GraphPad Software Inc., USA). Statistical significance of individual proteins was tested with independent *t* test followed by the Benjamini–Hochberg procedure (α = 0.05) to account for false-positive discoveries. One-way ANOVA followed by pairwise independent *t* test with Bonferroni correction were used for testing statistical significance in prostanoid concentration, fatty acid composition of total lipids, phospholipid profile, and sphingolipid concentration. The level of significance was set to *p* < 0.05. Principal component analysis (PCA) was performed on the lipidomics data (excluding the prostanoid data) using SIMCA P+ version 12 (MKS Data Analytics Solution, Umeå, Sweden).

## Results

### Effect of CIII and NS-398 on Prostanoid Profiles

The protein expressions of mPGES-1 and COX-2 were induced with IL-1β in the presence or absence of CIII or NS-398 during 24 h of incubation (**Figure 1B**). CIII decreased PGE_2_ production and increased PGF_2α_ and TXB_2_ formation while NS-398 blocked the production of all prostanoids (**Figure 1C**).

### Effects of CIII and NS-398 on Protein Profiles and Molecular Pathways

**Figure 2:**
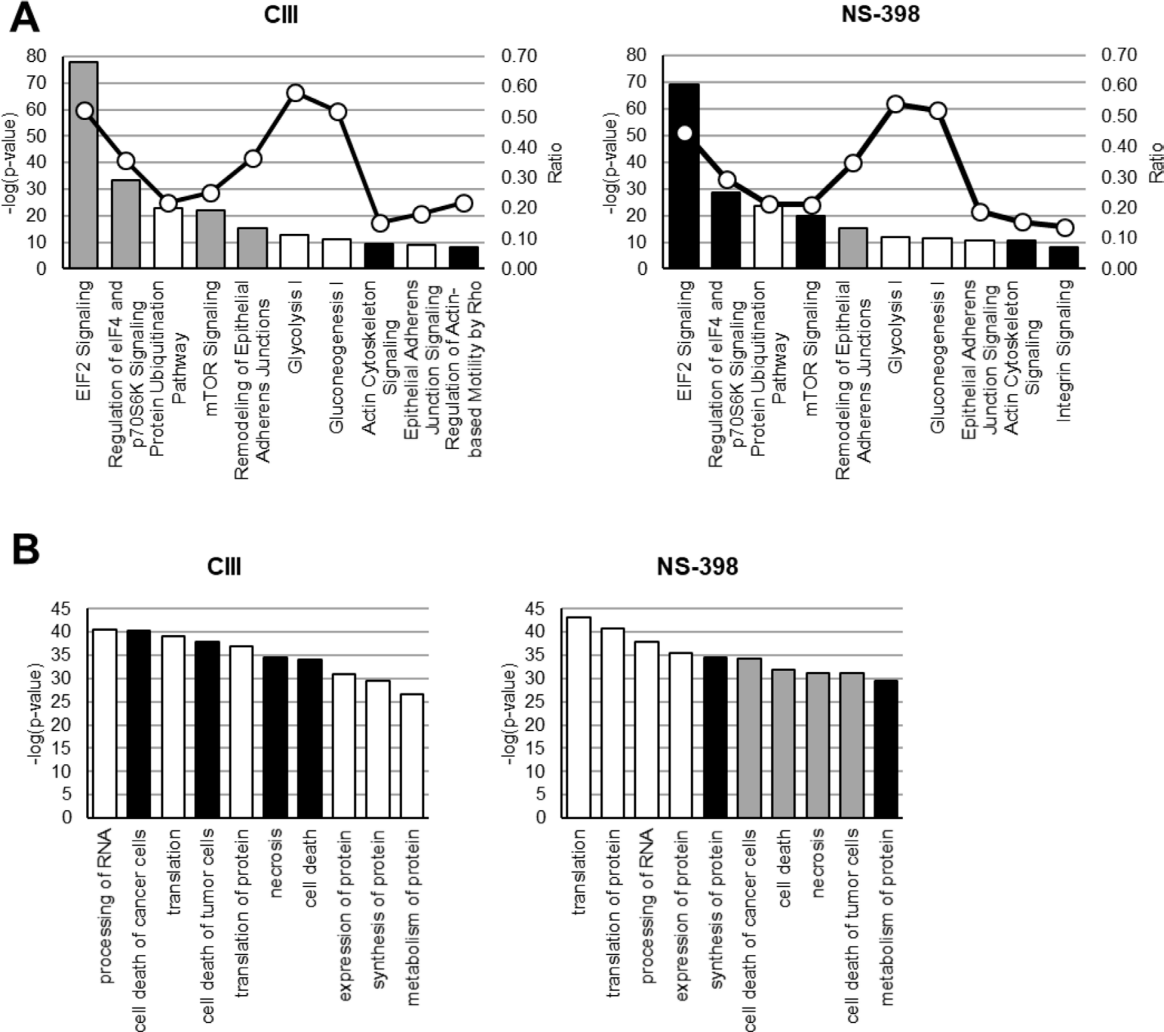
Effect on molecular functions by CIII and NS-398. IPA was performed on quantified proteins from CIII and NS-398 samples. **(A)** The top 10 affected canonical pathways based on *p* value are shown for each inhibitor. The calculated *Z* score indicates a pathway with proteins exhibiting overall increased levels (*Z* > 0, black bars) or decreased levels (*Z* < 0, gray bars). White bars represent no overall increase or decrease of proteins in the assigned pathway. The ratio (white dots connected by a black line) shows the fraction of proteins from the dataset that map to the pathway compared to the total number of proteins that map to the same pathway in the database. **(B)** The top 10 affected disease and function annotations for each inhibitor based on *p* value are shown. The different bar colors represent if a function is predicted to be increased in activation (*Z* > 2.0, black bars) or decreased in activation (*Z* < −2.0, gray bars). White bars represent change in function but the direction was not predicted. *Z* scores are presented in Results Section.

Quantitative LC-MS/MS-based proteomics was performed to analyze differences in protein levels in an untargeted manner. Subcellular fractionation and HPLC-based reversed-phase separation at peptide level were employed to achieve deep proteome coverage and stable isotope labeling of peptides was utilized for relative protein quantification. The total number of quantified proteins at the significant level (*p* < 0.01) across the dataset in the cytosolic and microsomal fractions was 860 and 882, respectively. The shared protein identities between the different treatment groups are shown in **Supplementary Figure S1**. The top 10 altered proteins by each inhibitor are presented in **Table 1**. Performing Benjamin–Hochberg procedure to account for multiple testing resulted in nine proteins for CIII and two proteins for NS-398 being altered compared to IL-1β treatment. Treatment with CIII increased Ubiquitin thioesterase OTUB1 (*p* = 0.00014) and Myosin light polypeptide 6 (*p* = 0.00014) with decreased Autism susceptibility gene 2 protein (*p* = 1.6E−11), Heterogenous nuclear ribonucleoprotein C-like 2 (*p* = 6.2E−05), L-aminoadipate-semialdehyde  dehydrogenase-phospho-pantetheinyl transferase (*p* = 0.00013), ATP-dependent DNA helicase Q1 (*p* = 0.00016), 4F2 cell-surface antigen heavy chain (*p* = 0.00021), Nucleolar protein 56 (*p* = 0.00024), and Pescadillo homolog (*p* = 0.00024). These proteins were not altered by IL-1β alone (**Supplementary Table S1**). Treatment with NS-398 decreased 60S ribosomal protein L21 (*p* = 2.5E−09) and TGF-beta receptor type-2 (*p* = 5.1E−06). However, only the effect in 60S ribosomal protein L21 was unique for NS-398 as the change in TGF-beta receptor type-2 was also observed with IL-1β alone (**Supplementary Table S1**). Pathway analysis applied to the entire dataset revealed that the most affected canonical pathways based on *p* value were the same for both inhibitors (**Figure 2A**). EIF2 signaling, regulation of eIF4 and p70S6K signaling, and mTOR signaling were decreased with CIII treatment and increased with NS-398 treatment. Both inhibitors increased actin cytoskeletal signaling and decreased remodeling of epithelial adherens junctions. Disease and functions analysis predicted that functions belonging to the categories protein synthesis and cell death were the most affected by both inhibitors (**Figure 2B**). CIII treatment was predicted to increase cell death of cancer cells (*Z* = 3.8, *p* = 5.1E−41), cell death of tumor cells (*Z* = 3.7, *p* = 1.6E−38), necrosis (*Z* = 3.4, *p* = 2.7E−35), and cell death (*Z* = 3.7, *p* = 7.8E−35). NS-398 treatment was predicted to increase synthesis of proteins (*Z* = 2.5, *p* = 2.4E−35) and metabolism of proteins (*Z* = 2.9, 2.5E−30) and decrease cell death of cancer (*Z* = −5.0, *p* = 6.5E−35), cell death (*Z* = −4.6, *p* = 1.3E−32), necrosis (*Z* = −4.4, *p* = 5.9E−32), and cell death of tumor cells (*Z* = −5.0, *p* = 8.3E−32).

**Table 1:** Effect on protein profile by CIII and NS-398.

Top 10 proteins altered by CIII				
UniProt ID	Protein name	Fraction	Fold-change to Ctrl	*p* value
Q8WXX7	**Autism susceptibility gene 2 protein**	Microsomal	0.05	1.6E−11*
B2RXH8	**Heterogenous nuclear ribonucleoprotein C-like 2**	Cytosolic	0.57	6.2E−05*
Q9NRN7	**L-aminoadipate-semialdehyde dehydrogenase**	Microsomal	0.06	0.00013*
Q96FW1	**Ubiquitin thioesterase OTUB1**	Microsomal	1.61	0.00014*
P60660	**Myosin light polypeptide 6**	Cytosolic	1.74	0.00014*
P46063	**ATP-dependent DNA helicase Q1**	Microsomal	0.70	0.00016*
P08195	**4F2 cell-surface antigen heavy chain**	Microsomal	0.51	0.00021*
O00567	**Nucleolar protein 56**	Microsomal	0.47	0.00024*
O00541	**Pescadillo homolog**	Microsomal	0.69	0.00024*
Q9Y5B9	FACT complex subunit SPT16	Microsomal	0.45	0.00035
Top 10 proteins altered by NS-398				
UniProt ID	Protein name	Fraction	Fold-change to Ctrl	*p* value
P46778	**60S ribosomal protein L21**	Microsomal	0.004	2.5E−09*
P37173	TGF-beta receptor type-2	Cytosolic	0.03	5.1E−06*
Q6PUV4	Complexin-2	Cytosolic	0.08	0.00031
O43390	Heterogeneous nuclear ribonucleoprotein R	Cytosolic	0.77	0.0022
A6NCN2	Putative keratin-87 protein	Cytosolic	1.94	0.0042
P19388	DNA-directed RNA polymerases I, II, and III subunit RPABC1	Microsomal	0.17	0.0075
P48444	Coatomer subunit delta	Cytosolic	0.17	0.0077
P07237	Protein disulfide-isomerase	Microsomal	0.19	0.012
O14795	Protein unc-13 homolog B	Cytosolic	0.19	0.013
O60701	UDP-glucose 6-dehydrogenase	Cytosolic	1.26	0.013

Proteomics was performed on A549 cell pellets using LC-MS/MS. The top 10 proteins altered by CIII or NS-398 based on p value are listed. Statistical significance was tested using individual t test (n = 3) followed by the Benjamini–Hochberg procedure (*α* = 0.05). An asterisk (*) denotes significant p value after the Benjamini–Hochberg procedure. Proteins that were uniquely altered by inhibitors and not altered by IL-1*β* are highlighted in bold. The top altered proteins by IL-1*β* is shown in **Supplementary Table S1**.

### Effect of CIII and NS-398 on Lipid Profiles

**Figure 3:**
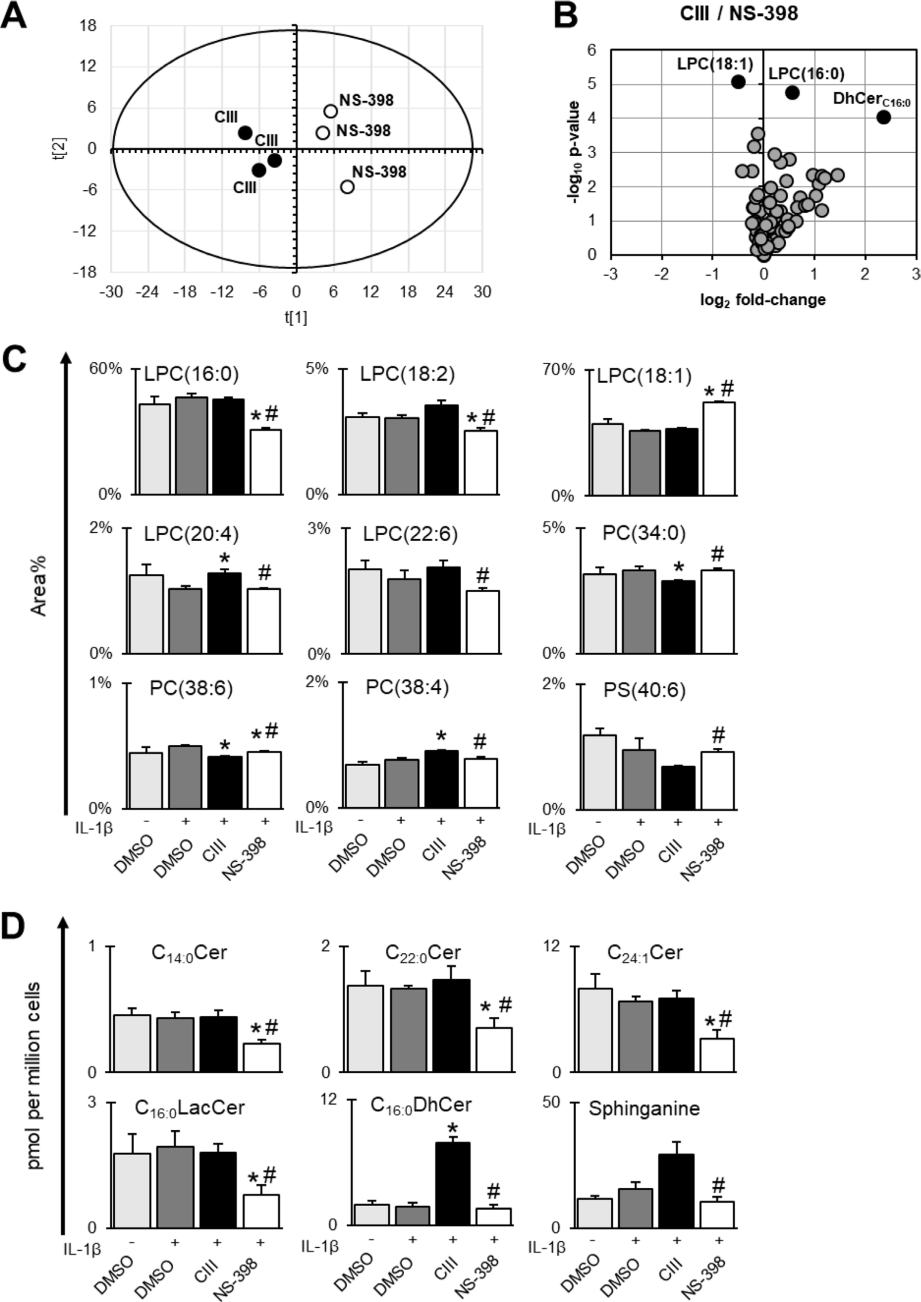
Differences between CIII and NS-398 based on lipidomics data. Fatty acids, phospholipids, and sphingolipids were measured in A549 cell pellets. **(A)** Score plot from unsupervised clustering by PCA showing separation of CIII and NS-398. The corresponding loadings plot is found in **Supplementary Figure S2**. **(B)** Volcano plot showing differences in lipids between CIII and NS-398. The top 3 altered lipid species based on *p* value (individual *t* test) are highlighted in black dots. **(C)** Differences between CIII and NS-398 in individual phospholipids expressed in area% (mean ± SD, *n* = 3) within each phospholipid class of one experiment. **(D)** Differences between CIII and NS-398 in individual sphingolipids expressed in pmol per million cells (mean ± SD, *n* = 3) for one experiment. Statistical significance was tested using ANOVA followed by individual *t* test with Bonferroni correction (*p* < 0.05). The asterisk (*) represents significance to IL-1β and the hashtag (#) represents significance between the two inhibitors. Complete datasets for lipidomics results are presented in **Supplementary Tables S2** to **S4**.

Multiple chromatography-based methods were used to quantify changes in the A549 cellular lipidome upon inhibition of mPGES-1 or COX-2. First, we detected and quantified 8 out of 23 measured fatty acids in our GC-FID analysis and there were no changes across the different treatments (**Supplementary Table S2**). Second, we detected and quantified 60 out of 107 phospholipids across the samples (**Supplementary Table S3**). Third, we detected and quantified 19 out of 25 measured sphingolipids across the samples (**Supplementary Table S4**). Multiple differences in lipid species between CIII and NS-398 were found (**Figure 3**). PCA was performed on the lipidomics data for CIII and NS-398 generated from these three platforms. The obtained model [R^2^X(1) = 0.51, R^2^X(2) = 0.18] showed separate clustering of the two inhibitors in a scores plot (**Figure 3A**). The corresponding loadings plot is shown in **Supplementary Figure S2**. The lipids most important for the separation were LPCs and ceramides. We found nine phospholipids to be significantly altered between the two inhibitors (**Figure 3C**). The strongest effect was observed in the LPC profile, where LPC(16:0), LPC(18:2), LPC(18:1), LPC(20:4), and LPC(22:6) were altered between the two inhibitors. Additionally, PC(38:4) was increased while PC(34:0), PC(38:6), and PS(40:6) were decreased with CIII treatment compared to NS-398. As for sphingolipids, treatment with CIII increased the concentration of sphinganine and C_16:0_DhCer while treatment with NS-398 resulted in a general decreased concentration in multiple sphingolipids: C_14:0_–Cer, C_22:0_–Cer, C_24:1_–Cer, and C_16:0_–LacCer (**Figure 3D**).

### Effect of CIII and NS-398 on Cell Death

**Figure 4:**
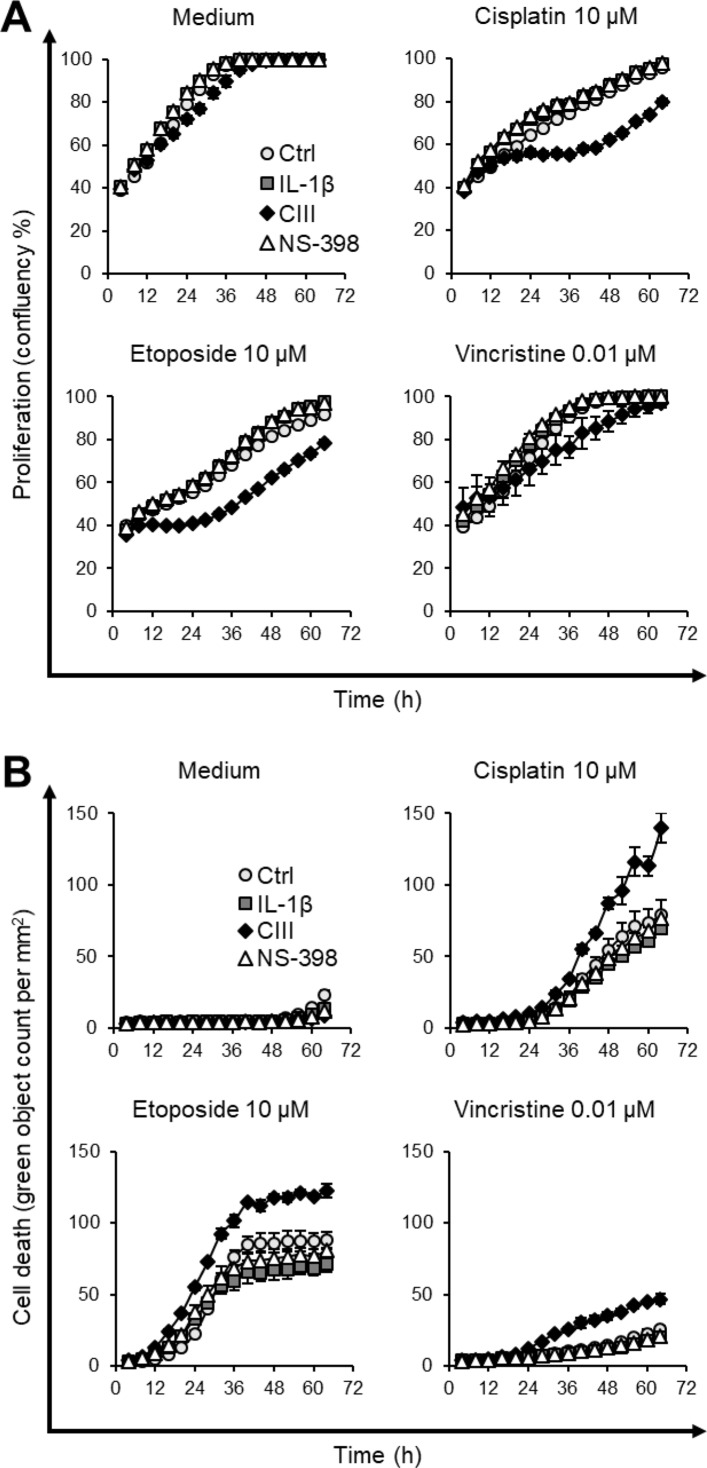
Effect on cell proliferation and cell death by CIII and NS-398 in combination with cytostatic drugs. **(A)** Measurement of cell proliferation by confluency after co-treatment with cisplatin, etoposide, or vincristine. **(B)** Quantification of cell death by SYTOX Green after co-treatment with cisplatin, etoposide, or vincristine. Data are presented as mean ± SD (*n* = 3) with Ctrl (light gray circle, ○), IL-1β (dark gray square, ▪), CIII (black diamond, ♦), and NS-398 (white triangle, Δ) from one experiment. The experiment was performed twice in triplicate. The graphs illustrate concentrations of cytostatic drugs where CIII increased the cytotoxic effect of cytostatic drugs. All tested concentrations and the two independent experiments are presented in **Supplementary Figure S4 **and **S5**.

The effect on cell death was investigated using the live cell imaging system IncuCyte. We first analyzed the effect of CIII and NS-398 on apoptosis. This was performed at high density seeding to mimic the omics experiments, and the cells were incubated with SYTOX Green for measuring cell death and fluorescent probes for PS exposure (annexin V) and active caspase-3/7 for measuring apoptosis. We did not observe any significant changes in cell death between the two inhibitors or compared to IL-1β alone (**Supplementary Figure S3**). We next investigated the effect of CIII and NS-398 on proliferation and whether the inhibitors altered the cytotoxicity of cytostatic drugs. The cells were seeded at lower density to enable monitoring for a longer time before the cells reached confluency. Our results show that CIII alone decreased the proliferation rate without inducing cell death (**Figure 4**; **Supplementary Figure S4** and **S5**). CIII potentiated the cytotoxicity of cisplatin (10 µM), etoposide (10 µM), and vincristine (0.01 µM), resulting in slower proliferation rate and increased cell death. NS-398 did not alter the cytotoxicity of the tested cytostatic drugs.

## Discussion

PGE_2_ is a key lipid mediator in inflammation and tumorigenesis, affecting virtually all aspects of cancer progression (Wang and Dubois, 2010). Reducing PGE_2_ concentration in the tumor microenvironment by selective inhibition of mPGES-1 is anticipated as an alternative strategy for anti-cancer treatment with improved selectivity and safety compared to conventional NSAIDs (Larsson and Jakobsson, 2015). In this study, we used proteomics and lipidomics platforms to investigate differences between mPGES-1 inhibitor CIII and COX-2 inhibitor NS-398 in IL-1β-induced A549 cells.

Selective mPGES-1 inhibitor CIII concentration-dependently reduces PGE_2_ production (Leclerc et al., 2013). This effect is in contrast to treatment with the selective COX-2 inhibitor NS-398, which inhibits the production of all prostanoids. We showed that inhibition of mPGES-1 in A549 cells also resulted in increased formation of PGF_2α_ and TXB_2_. The increase in these prostanoids could be explained by shunting of PGH_2_ to other terminal synthases when mPGES-1 is blocked, an effect that has been observed in studies on genetic deletion (Kapoor et al., 2006; Brenneis et al., 2008) and pharmacological inhibition (Beales and Ogunwobi, 2010; Ozen et al., 2017) of mPGES-1. In particular, increased PGF_2α_ formation has been reported for the mPGES-1 inhibitor MF63 in A549 cells (Xu et al., 2008). However, the effect on other prostanoids may be mediated by mechanisms other than redirection or shunting such as drug off-target effects or feedback loop effects.

While the detrimental roles of PGE_2_ are well established in multiple cancer forms, the contribution of PGF_2α_ and TXA_2_ is less studied. Results from *in vitro* studies showed that PGF_2α_ can stimulate migration and invasion of colon (Qualtrough et al., 2007) and endometrial (Keightley et al., 2010) cancer cells, where PGF_2α_ has been shown to induce proliferation (Sales et al., 2004) and angiogenesis (Sales et al., 2005) in endometrial cancer cell lines. These studies were conducted at ≥100 nM (= 35,000 pg/mL) PGF_2α_ that is well above expected physiological concentration and calculated affinities for prostanoid receptors (Abramovitz et al., 2000), but the effect of added PGF_2α_ was attenuated with the use of PGF_2α_ receptor (FP) antagonist (Keightley et al., 2010; Sales et al., 2005). It has been shown *in vivo* that stable expression of the FP receptor on endometrial cancer cells in a xenograft model increases neutrophil infiltration, but this does not influence tumor growth (Wallace et al., 2009). NSCLC tissues have increased expression of mPGES-1 (Yoshimatsu et al., 2001) and thromboxane synthase (TXAS) (Cathcart et al., 2011). TXA_2_ is a potent mediator of platelet aggregation and vasoconstriction, but it is also linked to tumor cell proliferation and invasion (Ekambaram et al., 2011). A549 cells express the FP receptor (Fang et al., 2004) and both spliced variants of the TXA_2_ receptor (TPα and TPβ) (Huang et al., 2013), meaning these prostanoids can act in an autocrine fashion.

It is difficult to extrapolate our prostanoid results *in vitro *to the outcome of mPGES-1 inhibition in a lung tumor *in vivo*, even when comparing with aforementioned literature. We and others have demonstrated the feasibility of mPGES-1 as a therapeutic target in cancer, as genetic deletion or pharmacological inhibition of mPGES-1 results in slower-growing tumors *in vivo* (Hanaka et al., 2009; Kamei et al., 2009; Nakanishi et al., 2011; Sasaki et al., 2012; Howe et al., 2013; Takahashi et al., 2014; Zelenay et al., 2015; Kock et al., 2018). Possible shunting of PGH_2_ in the tumor microenvironment when mPGES-1 is blocked is a potential concern, but it may also lead to the formation of prostanoids other than PGF_2α_ and TXB_2_ when diverse cells are present. For example, this could, in theory, lead to the generation of stromal-derived prostacyclin or mast cell-derived PGD_2_, where prostacyclin analog Iloprost (Nemenoff et al., 2008) and PGD_2_ analog BW245C (Murata et al., 2011) have shown anti-cancer effects in lung cancer mouse models. Indeed, studies on human vessels (Ozen et al., 2017) suggest that redirection of PGH_2_ to prostacyclin is the dominant effect *in vivo*. The only phase I clinical trial published with an mPGES-1 inhibitor in man (Jin et al., 2016) supports this concept, as a decreased amount of urinary PGE_2_ metabolite coincides with an increased amount of urinary prostacyclin metabolite. We conclude that selective reduction in PGE_2_ production upon mPGES-1 inhibition is key for anti-cancer properties and that the consequence(s) of the possible increase in other prostanoids needs to be evaluated *in vivo*.

The effect of mPGES-1 inhibitors on proteomic changes has not been reported in the literature. However, studies to investigate the effect of COX inhibitors on a proteomic scale in different cells have been performed (Lou et al., 2006; Baek et al., 2010; O’Brien et al., 2011; Rezaie et al., 2011). These studies collectively show that COX inhibitors can alter the proteome in different ways and sometimes in opposing direction depending on multiple parameters including inhibitor type, inhibitor concentration, treatment duration, cell type (origin and COX expression levels), and potential use of pro-inflammatory stimuli. Our proteomics analysis showed that treatment with CIII or NS-398 resulted in distinct changes in several proteins. CIII increased Ubiquitin thioesterase OTUB1 and Myosin light polypeptide 6 while it decreased Autism susceptibility gene 2 protein, Heterogenous nuclear ribonucleoprotein C-like 2, L-aminoadipate-semialdehyde dehydrogenase, ATP-dependent DNA helicase Q1, 4F2 cell-surface antigen heavy chain, Nucleolar protein 6, and Pescadillo homolog. Ubiquitin thioesterase OTUB1 regulates multiple pathways in cancer progression and increased expression of OTUB1 is associated with poor prognosis in several cancers including lung cancer (Saldana et al., 2019). Myosin light polypeptide 6 is a component of macromolecular complex myosin, and it is involved in the pathways ERK signaling and cytoskeleton remodeling *via* actin. Autism susceptibility gene 2 protein is implicated in neurodevelopment, and deletion of this gene has been associated with lung adenocarcinoma (Weir et al., 2007). Heterogenous nuclear ribonucleoprotein C-like 2 is a component of the 40S ribosomal subunit and is involved in RNA processing. L-aminoadipate-semialdehyde dehydrogenase is implicated in lysine metabolism. ATP-dependent DNA helicase Q1 may be involved in repair of DNA. 4F2 cell-surface antigen heavy chain is a transmembrane protein that is implicated in integrin signaling. Nuclear protein 6 is involved in rRNA biogenesis and its expression is associated with poorer prognosis in liver and thyroid cancer (data available from the Human Protein Atlas, HPA, version 18, www.proteinatlas.org). Pescadillo homolog is an estrogen-induced protein implicated in breast cancer development and progression (Li et al., 2009; Cheng et al., 2012). Treatment with NS-398 decreased 60S ribosomal protein L21, which is implicated in protein synthesis. Our data suggested differences in the abundance of individual proteins when treating cells with CIII or NS-398.

We acknowledge that it is difficult to extrapolate the biological significance of these individual protein changes in lung cancer based on proteomics data alone as this would require validation with orthogonal techniques (such as Western blot) and the role of each protein to be tested in functional assays. It should be pointed out that the inhibitors may influence enzyme activity *via* phosphorylation rather than protein abundances. Using a more translational approach, we performed IPA on the whole protein lists, which revealed that the two inhibitors had opposite effect on top canonical pathways including EIF2 signaling, regulation of eIF4 and p70S6K signaling, and mTOR signaling. These pathways are important for cell growth by controlling mRNA translation, autophagy, and metabolism (Guertin and Sabatini, 2007). The mTOR signaling pathway is dysregulated in many forms of cancer including NSCLC (Ekman et al., 2012) and the mTOR inhibitor rapamycin decreases A549 cell proliferation, induces autophagy, and sensitizes the cells to radiation (Li et al., 2016). The downregulation in these canonical pathways by CIII suggests an inhibitory effect on protein translation and alteration in lipid metabolism. Using IPA to annotate these changes to disease and cellular functions, we found that the predicted outcome of CIII treatment was promotion of cell death while NS-398 treatment was predicted to result in an anti-cell death state.

We used lipidomics platforms to investigate further differences between CIII and NS-398. Membrane lipid saturation is a shared feature among cancer cells, and this results in reduced membrane fluidity and dynamics, which ultimately is associated with increased oxidative stress tolerance and increased chemotherapy resistance (Beloribi-Djefaflia et al., 2016; Rohrig and Schulze, 2016). Using GC-FID analysis, we quantified fatty acid profiles of total lipids and there was no effect from the inhibitors, suggesting that neither inhibitor alters membrane fluidity. Moreover, we performed targeted LC-MS/MS analysis to measure cellular phospholipids. The strongest effect was seen in the LPC species, where LPC(18:1) was increased and LPC(16:0) and LPC(18:2) were decreased with NS-398. LPCs are mainly generated by metabolism of membrane PCs by cPLA_2_ (Burke and Dennis, 2009), and these lipids have been found to be involved in several cellular processes and sometimes with opposing effect depending on degree of saturation, concentration, and biological context (Sevastou et al., 2013; Drzazga et al., 2014). We conclude that inhibition of COX-2 caused a stronger effect in the LPC profile compared to inhibition of mPGES-1, where the biological consequence remains to be elucidated.

Furthermore, our sphingolipid analysis showed that CIII treatment increased the concentration of sphinganine and C_16:0_DhCer while NS-398 treatment resulted in a general reduction in ceramides. These changes can be linked to alterations reported in the literature. Schiffman *et al.* found that the anti-proliferative effect of the selective COX-2 inhibitor celecoxib in A549, HeLa, and HCT-116 cells was due to COX-2 independent alterations in sphingolipid levels, i.e., increase in sphinganine and DhCers (C_16:1_, C_24:1_, and C_24:0_) with decrease in Cers (C_24:1_ and C_24:0_) (Schiffmann et al., 2009). They concluded that these changes were due to both activation of *de novo* sphingolipid synthesis and inhibition of dihydroceramide desaturase (DEGS). This effect was unrelated to COX-2 inhibition since other tested COX-2 inhibitors did not alter sphingolipid levels. In line with this, several studies have shown that accumulation of DhCers precedes apoptosis in cancer cell lines. Jiang *et al.* have demonstrated this effect for vitamin E forms of γ-tocopherol in A549 cells (Jiang et al., 2004) and γ-tocotrienol in prostate cancer cell lines (Jiang et al., 2012), while Wang *et al.* have shown this effect for the anti-cancer agent fenretinide (4-hydroxyphenyl retinamide, 4-HPR) in an ovarian cancer cell line (Wang et al., 2008). Also, oxidative stress has been shown to lead to accumulation of DhCers *via* inhibition of DEGS without affecting the level of Cers in A549 cells (Idkowiak-Baldys et al., 2010). Cers are considered as tumor suppressors that can potentiate cell death and autophagy responses (Young et al., 2013). In particular, Cers can activate apoptosis *via* lysosomal effects (cleavage of Bid) or inhibition of anti-apoptotic mediators Bcl-2 and Bcl-XL, and activate JNK signaling and suppress Akt signaling that ultimately decreases cell survival. The reduction in ceramides together with activation of mTOR suggest increased cell survival or less apoptosis with NS-398 treatment. However, the molecular effect of Cers is dependent on acyl chain length and saturation degree (Ponnusamy et al., 2010). Collectively, our sphingolipids data pointed towards induction of cell death with CIII treatment compared to NS-398 treatment.

We finally investigated the effect of the inhibitors on cell death. CIII or NS-398 alone did not induce cell death in general or apoptosis specifically. When co-treating cells with the inhibitors and cytostatic drugs, we found that CIII increased the cytotoxicity of cisplatin, etoposide, and vincristine. We observed that CIII potentiated the anti-proliferative effect that coincides with increased induction of cell death. This is in line with previous observations that genetic knock-down or pharmacological inhibition of mPGES-1 increased the effect of the EGFR inhibitor erlotinib in prostate cancer cells *in vitro* and *in vivo* (Finetti et al., 2015). Multiple studies have reported that NSAIDs induce apoptosis or reduce proliferation *in vitro*, e.g., the use of celecoxib (Liu et al., 2004; Schiffmann et al., 2009; Ramer et al., 2013), NS-398 (Qiu et al., 2012), and indomethacin (Kato et al., 2011; Mandegary et al., 2013) in A549 cells. However, these studies were conducted at high (50–400 µM) NSAID concentrations, and the anti-cancer effect of especially celecoxib is not COX-2 exclusive (Schiffmann et al., 2009). Nonetheless, NSAIDs can potentiate the effect of cytostatic drugs *in vitro* (Duffy et al., 1998) and NSAIDs have been demonstrated to enhance the effect of checkpoint inhibitors in xenograft mouse models (Zelenay et al., 2015). Our results support that an mPGES-1 inhibitor would replicate these actions of NSAIDs by selectively lowering oncogenic PGE_2_ in the tumor microenvironment. However, our analysis showed that NS-398 decreased rather than increased cell death, despite reducing the production of PGE_2_. As discussed above, the cytotoxic effect upon mPGES-1 inhibition may be dependent on a decrease in PGE_2_ with a simultaneous increase in other prostanoids.

The limitation of our study is the use of a single inhibitor for mPGES-1 or COX-2 at one concentration. The concentrations were selected based on each inhibitor’s potency to suppress the induced PGE_2_ production to a similar level in this particular setting (Leclerc et al., 2013), and the reduction in PGE_2_ was evaluated in each experiment in our current study. We acknowledge that our experiments were performed on one cell line, where A549 is regarded as a good model system to study the COX/mPGES-1/PGE_2_ pathway. We encourage additional investigations with other inhibitors and cell-based systems to confirm the observed changes.

In conclusion, we report that selective inhibition of mPGES-1 or COX-2 affects protein and lipid profiles differently in A549 cells. Our proteomics and lipidomics data suggested a pro-cell death state with inhibition of mPGES-1 compared to inhibition of COX-2 based on 1) changes in molecular pathways based on alterations in protein profiles and 2) accumulation in two sphingolipid species (C_16:0_DhCer and sphinganine) previously described in cancer cell death. The change in cell death was demonstrated in live cell imaging experiments, where CIII decreased proliferation and potentiated the cytotoxic effect of cisplatin, etoposide, and vincristine. Hence, inhibition of mPGES-1 can sensitize cancer cells to treatment with cytotoxic drugs, and this could be a valid therapeutic strategy to improve efficacy of existing chemotherapy. Results from our study motivate the development of selective mPGES-1 inhibitors as therapeutic adjuvants to treat cancer.

## Data Availability Statement

The datasets generated for this study can be found in PRIDE, PXD013490.

## Author Contributions

Study concept and design was done by FB, EO, HI, KL, MK, and P-JJ. Acquisition of data was done by FB, EO, HI, JR, AC, and KE. Analysis and interpretation of data was performed by FB, EO, HI, AC, PK, KK, KL, MK, and P-JJ. Statistical analysis was performed by FB, EO, and HI. Drafting of the manuscript was done by FB, EO, HI, JR, and AC. Administrative, technical, or material support was performed by PE and CW. All authors critically revised and approved the final version of the manuscript.

## Funding

This work was supported by grants from the Swedish Research Council (grant no: 2017-02577), Innovative Medicines Initiative (EU/EFPIA, ULTRA-DD, grant no: 115766), Stockholm County Council (ALF, grant no: 20160378), the Swedish Rheumatism Association (grant no: R-755861), King Gustaf V’s 80 Years Foundation (grant no: n/a), the Swedish Cancer Society (grant no: CAN2016/739), the Cancer Society in Stockholm (grant no: 171073), and funds from Karolinska Institutet (grant no: n/a).

## Conflict of Interest Statement

P-JJ is member of the board of directors at Gesynta Pharma, a company that develops anti-inflammatory drugs. The remaining authors declare that the research was conducted in the absence of any commercial or financial relationships that could be construed as a potential conflict of interest.

## References

[B1] Abramovitz M. Adam M. Boie Y. Carriere M. Denis D. Godbout C. (2000). The utilization of recombinant prostanoid receptors to determine the affinities and selectivities of prostaglandins and related analogs. Biochim. Biophys. Acta. 1483 (2), 285–293. 10.1016/S1388-1981(99)00164-X 10634944

[B2] Baek S. M. Ahn J. S. Noh H. S. Park J. Kang S. S. Kim D. R. (2010). Proteomic analysis in NSAIDs-treated primary cardiomyocytes. J. Proteomics 73 (4), 721–732. 10.1016/j.jprot.2009.10.004 19850159

[B3] Beales I. L. Ogunwobi O. O. (2010). Microsomal prostaglandin E synthase-1 inhibition blocks proliferation and enhances apoptosis in oesophageal adenocarcinoma cells without affecting endothelial prostacyclin production. Int. J. Cancer 126 (9), 2247–2255. 10.1002/ijc.24875 19739114

[B4] Beloribi-Djefaflia S. Vasseur S. Guillaumond F. (2016). Lipid metabolic reprogramming in cancer cells. Oncogenesis 5, e189. 10.1038/oncsis.2015.49 26807644 PMC4728678

[B5] Billich A. Bornancin F. Mechtcheriakova D. Natt F. Huesken D. Baumruker T. (2005). Basal and induced sphingosine kinase 1 activity in A549 carcinoma cells: function in cell survival and IL-1beta and TNF-alpha induced production of inflammatory mediators. Cell Signal 17 (10), 1203–1217. 10.1016/j.cellsig.2004.12.005 16038795

[B6] Blaine S. A. Meyer A. M. Hurteau G. Wick M. Hankin J. A. Murphy R C. (2005). Targeted over-expression of mPGES-1 and elevated PGE2 production is not sufficient for lung tumorigenesis in mice. Carcinogenesis 26 (1), 209–217. 10.1093/carcin/bgh302 15471895

[B7] Boersema P. J. Raijmakers R. Lemeer S. Mohammed S. Heck A. J. (2009). Multiplex peptide stable isotope dimethyl labeling for quantitative proteomics. Nat. Protoc. 4 (4), 484–494. 10.1038/nprot.2009.21 19300442

[B8] Brenneis C. Coste O. Schmidt R. Angioni C. Popp L. Nusing R. M. (2008). Consequences of altered eicosanoid patterns for nociceptive processing in mPGES-1-deficient mice. J. Cell. Mol. Med. 12 (2), 639–648. 10.1111/j.1582-4934.2007.00110.x 18419601 PMC3822549

[B9] Burke J. E. Dennis E. A. (2009). Phospholipase A2 structure/function, mechanism, and signaling. J. Lipid Res. 50 Suppl, S237–S242. 10.1194/jlr.R800033-JLR200 19011112 PMC2674709

[B10] Cancer Genome Atlas Research, N (2014). Comprehensive molecular profiling of lung adenocarcinoma. Nature 511 (7511), 543–550. 10.1038/nature13385 25079552 PMC4231481

[B11] Cathcart M. C. Gately K. Cummins R. Kay E. O’Byrne K. J. Pidgeon G. P. (2011). Examination of thromboxane synthase as a prognostic factor and therapeutic target in non-small cell lung cancer. Mol. Cancer 10, 25. 10.1186/1476-4598-10-25 21388528 PMC3074522

[B12] Chambers M. C. Maclean B. Burke R. Amodei D. Ruderman D. L. Neumann S. (2012). A cross-platform toolkit for mass spectrometry and proteomics. Nat. Biotechnol. 30 (10), 918–920. 10.1038/nbt.2377 23051804 PMC3471674

[B13] Checa A. Khademi M. Sar D. G. Haeggstrom J. Z. Lundberg J. O. Piehl F. (2015). Hexosylceramides as intrathecal markers of worsening disability in multiple sclerosis. Mult. Scler. 21 (10), 1271–1279. 10.1177/1352458514561908 25480867

[B14] Cheng L. Li J. Han Y. Lin J. Niu C. Zhou Z. (2012). PES1 promotes breast cancer by differentially regulating ERalpha and ERbeta. J. Clin. Invest. 122 (8), 2857–2870. 10.1172/JCI62676 22820289 PMC3408741

[B15] Cheng Y. Wang M. Yu Y. Lawson J. Funk C. D. Fitzgerald G. A. (2006). Cyclooxygenases, microsomal prostaglandin E synthase-1, and cardiovascular function. J. Clin. Invest. 116 (5), 1391–1399. 10.1172/JCI27540 16614756 PMC1435722

[B16] Drzazga A. Sowinska A. Koziolkiewicz M. (2014). Lysophosphatidylcholine and lysophosphatidylinosiol—novel promissing signaling molecules and their possible therapeutic activity. Acta. Pol. Pharm. 71 (6), 887–899. 25745761

[B17] Duffy C. P. Elliott C. J. O’Connor R. A. Heenan M. M. Coyle S. Cleary I. M. (1998). Enhancement of chemotherapeutic drug toxicity to human tumour cells *in vitro* by a subset of non-steroidal anti-inflammatory drugs (NSAIDs). Eur. J. Cancer 34 (8), 1250–1259. 10.1016/S0959-8049(98)00045-8 9849488

[B18] Ekambaram P. Lambiv W. Cazzolli R. Ashton A. W. Honn K. V. (2011). The thromboxane synthase and receptor signaling pathway in cancer: an emerging paradigm in cancer progression and metastasis. Cancer Metastasis Rev. 30 (3-4), 397–408. 10.1007/s10555-011-9297-9 22037941 PMC4175445

[B19] Ekman S. Wynes M. W. Hirsch F. R. (2012). The mTOR pathway in lung cancer and implications for therapy and biomarker analysis. J. Thorac. Oncol. 7 (6), 947–953. 10.1097/JTO.0b013e31825581bd 22588151

[B20] Eriksson H. Lengqvist J. Hedlund J. Uhlen K. Orre L. M. Bjellqvist B. (2008). Quantitative membrane proteomics applying narrow range peptide isoelectric focusing for studies of small cell lung cancer resistance mechanisms. Proteomics 8 (15), 3008–3018. 10.1002/pmic.200800174 18654985

[B21] Fang K. M. Shu W. H. Chang H. C. Wang J. J. Mak O. T. (2004). Study of prostaglandin receptors in mitochondria on apoptosis of human lung carcinoma cell line A549. Biochem. Soc. Trans. 32 (Pt 6), 1078–1080. 10.1042/BST0321078 15506970

[B22] Finetti F. Terzuoli E. Giachetti A. Santi R. Villari D. Hanaka H. (2015). mPGES-1 in prostate cancer controls stemness and amplifies epidermal growth factor receptor-driven oncogenicity. Endocr. Relat. Cancer 22 (4), 665–678. 10.1530/ERC-15-0277 26113609 PMC4526795

[B23] Grosser T. Fries S. FitzGerald G. A. (2006). Biological basis for the cardiovascular consequences of COX-2 inhibition: therapeutic challenges and opportunities. J. Clin. Invest. 116 (1), 4–15. 10.1172/JCI27291 16395396 PMC1323269

[B24] Guertin D. A. Sabatini D. M. (2007). Defining the role of mTOR in cancer. Cancer Cell 12 (1), 9–22. 10.1016/j.ccr.2007.05.008 17613433

[B25] Hall Z. Ament Z. Wilson C. H. Burkhart D. L. Ashmore T. Koulman A. (2016). Myc expression drives aberrant lipid metabolism in lung cancer. Cancer Res. 76 (16), 4608–4618. 10.1158/0008-5472.CAN-15-3403 27335109

[B26] Hanaka H. Pawelzik S. C. Johnsen J. I. Rakonjac M. Terawaki K. Rasmuson A. (2009). Microsomal prostaglandin E synthase 1 determines tumor growth *in vivo* of prostate and lung cancer cells. Proc. Natl. Acad. Sci. U. S. A. 106 (44), 18757–18762. 10.1073/pnas.0910218106 19846775 PMC2768589

[B27] Harper K. A. Tyson-Capper A. J. (2008). Complexity of COX-2 gene regulation. Biochem. Soc. Trans. 36 (Pt 3), 543–545. 10.1042/BST0360543 18482003

[B28] Howe L. R. Subbaramaiah K. Kent C. V. Zhou X. K. Chang S. H. Hla T. (2013). Genetic deletion of microsomal prostaglandin E synthase-1 suppresses mouse mammary tumor growth and angiogenesis. Prostaglandins Other Lipid Mediat. 106, 99–105. 10.1016/j.prostaglandins.2013.04.002 23624019 PMC3830707

[B29] Huang R. Y. Li M. Y. Ng C. S. Wan I. Y. Kong A. W. Du J. (2013). Thromboxane A2 receptor alpha promotes tumor growth through an autoregulatory feedback pathway. J. Mol. Cell. Biol. 5 (6), 380–390. 10.1093/jmcb/mjt038 24115277

[B30] Idborg H. Olsson P. Leclerc P. Raouf J. Jakobsson P. J. Korotkova M. (2013). Effects of mPGES-1 deletion on eicosanoid and fatty acid profiles in mice. Prostaglandins Other Lipid Mediat. 107, 18–25. 10.1016/j.prostaglandins.2013.07.004 23916744

[B31] Idkowiak-Baldys J. Apraiz A. Li L. Rahmaniyan M. Clarke C. J. Kraveka J. M. (2010). Dihydroceramide desaturase activity is modulated by oxidative stress. Biochem. J. 427 (2), 265–274. 10.1042/BJ20091589 20105137 PMC3086801

[B32] Jakobsson P. J. Thoren S. Morgenstern R. Samuelsson B. (1999). Identification of human prostaglandin E synthase: a microsomal, glutathione-dependent, inducible enzyme, constituting a potential novel drug target. Proc. Natl. Acad. Sci. U. S. A. 96 (13), 7220–7225. 10.1073/pnas.96.13.7220 10377395 PMC22058

[B33] Jiang Q. Rao X. Kim C. Y. Freiser H. Zhang Q. Jiang Z. (2012). Gamma-tocotrienol induces apoptosis and autophagy in prostate cancer cells by increasing intracellular dihydrosphingosine and dihydroceramide. Int. J. Cancer 130 (3), 685–693. 10.1002/ijc.26054 21400505 PMC3381336

[B34] Jiang Q. Wong J. Fyrst H. Saba J. D. Ames B. N. (2004). gamma-Tocopherol or combinations of vitamin E forms induce cell death in human prostate cancer cells by interrupting sphingolipid synthesis. Proc. Natl. Acad. Sci. U. S. A. 101 (51), 17825–17830. 10.1073/pnas.0408340102 15596715 PMC535585

[B35] Jin Y. Smith C. L. Hu L. Campanale K. M. Stoltz R. Huffman L. G. Jr. (2016). Pharmacodynamic comparison of LY3023703, a novel microsomal prostaglandin e synthase 1 inhibitor, with celecoxib. Clin. Pharmacol. Ther. 99 (3), 274–284. 10.1002/cpt.260 26351780

[B36] Kalinski P. (2012). Regulation of immune responses by prostaglandin E2. J. Immunol. 188 (1), 21–28. 10.4049/jimmunol.1101029 22187483 PMC3249979

[B37] Kamei D. Murakami M. Sasaki Y. Nakatani Y. Majima M. Ishikawa Y. (2009). Microsomal prostaglandin E synthase-1 in both cancer cells and hosts contributes to tumour growth, invasion and metastasis. Biochem. J. 425 (2), 361–371. 10.1042/BJ20090045 19845504 PMC2825730

[B38] Kapoor M. Kojima F. Qian M. Yang L. Crofford L. J. (2006). Shunting of prostanoid biosynthesis in microsomal prostaglandin E synthase-1 null embryo fibroblasts: regulatory effects on inducible nitric oxide synthase expression and nitrite synthesis. FASEB J. 20 (13), 2387–2389. 10.1096/fj.06-6366fje 17023389 PMC4415996

[B39] Kato T. Fujino H. Oyama S. Kawashima T. Murayama T. (2011). Indomethacin induces cellular morphological change and migration *via* epithelial–mesenchymal transition in A549 human lung cancer cells: a novel cyclooxygenase-inhibition-independent effect. Biochem. Pharmacol. 82 (11), 1781–1791. 10.1016/j.bcp.2011.07.096 21840302

[B40] Keightley M. C. Sales K. J. Jabbour H. N. (2010). PGF2alpha-F-prostanoid receptor signalling *via* ADAMTS1 modulates epithelial cell invasion and endothelial cell function in endometrial cancer. BMC Cancer 10, 488. 10.1186/1471-2407-10-488 20840749 PMC2944181

[B41] Khuri F. R. Wu H. Lee J. J. Kemp B. L. Lotan R. Lippman S. M. (2001). Cyclooxygenase-2 overexpression is a marker of poor prognosis in stage I non-small cell lung cancer. Clin. Cancer Res. 7 (4), 861–867. 11309334

[B42] Kim S. Pevzner P. A. (2014). MS-GF+ makes progress towards a universal database search tool for proteomics. Nat. Commun. 5, 5277. 10.1038/ncomms6277 25358478 PMC5036525

[B43] Kock A. Larsson K. Bergqvist F. Eissler N. Elfman L. H. M. Raouf J. (2018). Inhibition of microsomal prostaglandin E synthase-1 in cancer-associated fibroblasts suppresses neuroblastoma tumor growth. EBioMedicine, 32, 84–92. 10.1016/j.ebiom.2018.05.008 29804818 PMC6021299

[B44] Larsson K. Jakobsson P. J. (2015). Inhibition of microsomal prostaglandin E synthase-1 as targeted therapy in cancer treatment. Prostaglandins Other Lipid Mediat. 120, 161–165. 10.1016/j.prostaglandins.2015.06.002 26100239

[B45] Leclerc P. Idborg H. Spahiu L. Larsson C. Nekhotiaeva N. Wannberg J. (2013). Characterization of a human and murine mPGES-1 inhibitor and comparison to mPGES-1 genetic deletion in mouse models of inflammation. Prostaglandins Other Lipid Mediat. 107, 26–34. 10.1016/j.prostaglandins.2013.09.001 24045148

[B46] Li J. Yu L. Zhang H. Wu J. Yuan J. Li X. (2009). Down-regulation of pescadillo inhibits proliferation and tumorigenicity of breast cancer cells. Cancer Sci. 100 (12), 2255–2260. 10.1111/j.1349-7006.2009.01325.x 19764998 PMC11159139

[B47] Li Y. Liu F. Wang Y. Li D. Guo F. Xu L. (2016). Rapamycin-induced autophagy sensitizes A549 cells to radiation associated with DNA damage repair inhibition. Thorac. Cancer 7 (4), 379–386. 10.1111/1759-7714.12332 27385978 PMC4930955

[B48] Liu X. Yue P. Zhou Z. Khuri F. R. Sun S. Y. (2004). Death receptor regulation and celecoxib-induced apoptosis in human lung cancer cells. J. Natl. Cancer Inst. 96 (23), 1769–1780. 10.1093/jnci/djh322 15572759

[B49] Lou J. Fatima N. Xiao Z. Stauffer S. Smythers G. Greenwald P. (2006). Proteomic profiling identifies cyclooxygenase-2-independent global proteomic changes by celecoxib in colorectal cancer cells. Cancer Epidemiol. Biomarkers Prev. 15 (9), 1598–1606. 10.1158/1055-9965.EPI-06-0216 16985019

[B50] Mandegary A. Torshabi M. Seyedabadi M. Amirheidari B. Sharif E. Ghahremani M. H. (2013). Indomethacin-enhanced anticancer effect of arsenic trioxide in A549 cell line: involvement of apoptosis and phospho-ERK and p38 MAPK pathways. Biomed. Res. Int. 2013, 237543. 10.1155/2013/237543 24312908 PMC3842073

[B51] Murata T. Aritake K. Matsumoto S. Kamauchi S. Nakagawa T. Hori M. (2011). Prostagladin D2 is a mast cell-derived antiangiogenic factor in lung carcinoma. Proc. Natl. Acad. Sci. U. S. A. 108 (49), 19802–19807. 10.1073/pnas.1110011108 22106279 PMC3241753

[B52] Nahnsen S. Bertsch A. Rahnenfuhrer J. Nordheim A. Kohlbacher O. (2011). Probabilistic consensus scoring improves tandem mass spectrometry peptide identification. J. Proteome. Res. 10 (8), 3332–3343. 10.1021/pr2002879 21644507

[B53] Nakanishi M. Menoret A. Tanaka T. Miyamoto S. Montrose D. C. Vella A. T. (2011). Selective PGE(2) suppression inhibits colon carcinogenesis and modifies local mucosal immunity. Cancer Prev. Res. (Phila) 4 (8), 1198–1208. 10.1158/1940-6207.CAPR-11-0188 21576350 PMC3151318

[B54] Nakanishi M. Rosenberg D. W. (2013). Multifaceted roles of PGE2 in inflammation and cancer. Semin. Immunopathol. 35 (2), 123–137. 10.1007/s00281-012-0342-8 22996682 PMC3568185

[B55] Nemenoff R. Meyer A. M. Hudish T. M. Mozer A. B. Snee A. Narumiya S. (2008). Prostacyclin prevents murine lung cancer independent of the membrane receptor by activation of peroxisomal proliferator-activated receptor gamma. Cancer Prev. Res. (Phila) 1 (5), 349–356. 10.1158/1940-6207.CAPR-08-0145 19138979 PMC2680197

[B56] Nishizawa N. Ito Y. Eshima K. Ohkubo H. Kojo K. Inoue T. (2018). Inhibition of microsomal prostaglandin E synthase-1 facilitates liver repair after hepatic injury in mice. J. Hepatol. 69 (1), 110–120. 10.1016/j.jhep.2018.02.009 29458169

[B57] O’BrienJ.HansenK.BarkanD.GreenJ.SchedinP.O’BrienJ. (2011). Non-steroidal anti-inflammatory drugs target the pro-tumorigenic extracellular matrix of the postpartum mammary gland. Int. J. Dev. Biol. 55 (7–9), 745–755. 10.1387/ijdb.113379jo 22161831

[B58] Ozen G. Gomez I. Daci A. Deschildre C. Boubaya L. Teskin O. (2017). Inhibition of microsomal PGE synthase-1 reduces human vascular tone by increasing PGI2: a safer alternative to COX-2 inhibition. Br. J. Pharmacol. 174 (22), 4087–4098. 10.1111/bph.13939 28675448 PMC5660006

[B59] Perez-Riverol Y. Csordas A. Bai J. Bernal-Llinares M. Hewapathirana S. Kundu D. J. (2019). The PRIDE database and related tools and resources in 2019: improving support for quantification data. Nucleic Acids Res. 47 (D1), D442–D450. 10.1093/nar/gky1106 30395289 PMC6323896

[B60] Petkova D. K. Clelland C. Ronan J. Pang L. Coulson J. M. Lewis S. (2004). Overexpression of cyclooxygenase-2 in non-small cell lung cancer. Respir. Med. 98 (2), 164–172. 10.1016/j.rmed.2003.09.006 14971881

[B61] Ponnusamy S. Meyers-Needham M. Senkal C. E. Saddoughi S. A. Sentelle D. Selvam S. P. (2010). Sphingolipids and cancer: ceramide and sphingosine-1-phosphate in the regulation of cell death and drug resistance. Future Oncol. 6 (10), 1603–1624. 10.2217/fon.10.116 21062159 PMC3071292

[B62] Qiu R. Chen J. Sima J. Shen X. Liu D. Shen J. (2012). NS398 induces apoptosis in non-small cell lung cancer cells. J. Cancer. Res. Clin. Oncol. 138 (1), 119–124. 10.1007/s00432-011-1080-3 22048655 PMC11824803

[B63] Qualtrough D. Kaidi A. Chell S. Jabbour H. N. Williams A. C. Paraskeva C. (2007). Prostaglandin F-2 alpha stimulates motility and invasion in colorectal tumor cells. Int. J. Cancer 121 (4), 734–740. 10.1002/ijc.22755 17437271 PMC2694992

[B64] Ramer R. Walther U. Borchert P. Laufer S. Linnebacher M. Hinz B. (2013). Induction but not inhibition of COX-2 confers human lung cancer cell apoptosis by celecoxib. J. Lipid Res. 54 (11), 3116–3129. 10.1194/jlr.M042283 23943857 PMC3793616

[B65] Raouf J. Idborg H. Englund P. Alexanderson H. Dastmalchi M. Jakobsson P. J. (2018). Targeted lipidomics analysis identified altered serum lipid profiles in patients with polymyositis and dermatomyositis. Arthritis. Res. Ther. 20 (1), 83. 10.1186/s13075-018-1579-y 29720222 PMC5932839

[B66] Rezaie F. Salimi M. Ghahremani M. H. Vaziri B. (2011). Potential molecular targets in chemopreventative action of celecoxib: a proteomics analysis of J774.A1 macrophage-like cell line. Mol. Biosyst. 7 (4), 1306–1311. 10.1039/c0mb00201a 21258746

[B67] Ricciotti E. FitzGerald G. A. (2011). Prostaglandins and inflammation. Arterioscler. Thromb. Vasc. Biol. 31 (5), 986–1000. 10.1161/ATVBAHA.110.207449 21508345 PMC3081099

[B68] Rohrig F. Schulze A. (2016). The multifaceted roles of fatty acid synthesis in cancer. Nat. Rev. Cancer. 16 (11), 732–749. 10.1038/nrc.2016.89 27658529

[B69] Rost H. L. Sachsenberg T. Aiche S. Bielow C. Weisser H. Aicheler F. (2016). OpenMS: a flexible open-source software platform for mass spectrometry data analysis. Nat. Methods 13 (9), 741–748. 10.1038/nmeth.3959 27575624

[B70] Saldana M. VanderVorst K. Berg A. L. Lee H. Carraway K. L. (2019). Otubain 1: a non-canonical deubiquitinase with an emerging role in cancer. Endocr. Relat. Cancer 26 (1), R1–R14. 10.1530/ERC-18-0264 30400005 PMC6226034

[B71] Sales K. J. List T. Boddy S. C. Williams A. R. Anderson R. A. Naor Z. (2005). A novel angiogenic role for prostaglandin F2alpha-FP receptor interaction in human endometrial adenocarcinomas. Cancer Res. 65 (17), 7707–7716. 10.1158/0008-5472.CAN-05-0101 16140938 PMC2694301

[B72] Sales K. J. Milne S. A. Williams A. R. W. Anderson R. A. Jabbour H. N. (2004). Expression, localization, and signaling of prostaglandin F-2 alpha receptor in human endometrial adenocarcinoma: regulation of proliferation by activation of the epidermal growth factor receptor and mitogen-activated protein kinase signaling pathways. J. Clin. Endocrinol. Metabol. 89 (2), 986–993. 10.1210/jc.2003-031434 14764825

[B73] Sasaki Y. Kamei D. Ishikawa Y. Ishii T. Uematsu S. Akira S. (2012). Microsomal prostaglandin E synthase-1 is involved in multiple steps of colon carcinogenesis. Oncogene 31 (24), 2943–2952. 10.1038/onc.2011.472 21986945

[B74] Schiffmann S. Sandner J. Schmidt R. Birod K. Wobst I. Schmidt H. (2009). The selective COX-2 inhibitor celecoxib modulates sphingolipid synthesis. J. Lipid. Res. 50 (1), 32–40. 10.1194/jlr.M800122-JLR200 18711209

[B75] Sevastou I. Kaffe E. Mouratis M. A. Aidinis V. (2013). Lysoglycerophospholipids in chronic inflammatory disorders: the PLA(2)/LPC and ATX/LPA axes. Biochim. Biophys. Acta. 1831 (1), 42–60. 10.1016/j.bbalip.2012.07.019 22867755

[B76] Smith W. L. Urade Y. Jakobsson P. J. (2011). Enzymes of the cyclooxygenase pathways of prostanoid biosynthesis. Chem. Rev. 111 (10), 5821–5865. 10.1021/cr2002992 21942677 PMC3285496

[B77] Sorensen H. T. Mellemkjaer L. Blot W. J. Nielsen G. L. Steffensen F. H. McLaughlin J. K. (2000). Risk of upper gastrointestinal bleeding associated with use of low-dose aspirin. Am. J. Gastroenterol. 95 (9), 2218–2224. 10.1111/j.1572-0241.2000.02248.x 11007221

[B78] Takahashi R. Amano H. Satoh T. Tabata K. Ikeda M. Kitasato H. (2014). Roles of microsomal prostaglandin E synthase-1 in lung metastasis formation in prostate cancer RM9 cells. Biomed. Pharmacother. 68 (1), 71–77. 10.1016/j.biopha.2013.10.008 24291175

[B79] Wallace A. E. Sales K. J. Catalano R. D. Anderson R. A. Williams A. R. Wilson M. R. (2009). Prostaglandin F2alpha-F-prostanoid receptor signaling promotes neutrophil chemotaxis *via* chemokine (C-X-C motif) ligand 1 in endometrial adenocarcinoma. Cancer Res. 69 (14), 5726–5733. 10.1158/0008-5472.CAN-09-0390 19549892 PMC2712458

[B80] Wang D. Dubois R. N. (2010). Eicosanoids and cancer. Nat. Rev. Cancer. 10 (3), 181–193. 10.1038/nrc2809 20168319 PMC2898136

[B81] Wang H. Maurer B. J. Liu Y. Y. Wang E. Allegood J. C. Kelly S. (2008). *N*-(4-Hydroxyphenyl)retinamide increases dihydroceramide and synergizes with dimethylsphingosine to enhance cancer cell killing. Mol. Cancer Ther. 7 (9), 2967–2976. 10.1158/1535-7163.MCT-08-0549 18790777

[B82] Weir B. A. Woo M. S. Getz G. Perner S. Ding L. Beroukhim R. (2007). Characterizing the cancer genome in lung adenocarcinoma. Nature 450 (7171), 893–898. 10.1038/nature06358 17982442 PMC2538683

[B83] Wessel D. Flügge U. I. (1984). A method for the quantitative recovery of protein in dilute solution in the presence of detergents and lipids. Anal. Biochem. 138 (1), 141–143. 10.1016/0003-2697(84)90782-6 6731838

[B84] Westman M. Korotkova M. Klint E. Stark A. Audoly L. P. Klareskog L. (2004). Expression of microsomal prostaglandin E synthase 1 in rheumatoid arthritis synovium. Arthritis. Rheum. 50 (6), 1774–1780. 10.1002/art.20286 15188353

[B85] Wu Y. C. Su L. J. Wang H. W. Jeff Lin C. F. Hsu W. H. Chou T. Y. (2010). Co-overexpression of cyclooxygenase-2 and microsomal prostaglandin E synthase-1 adversely affects the postoperative survival in non-small cell lung cancer. J. Thorac. Oncol. 5 (8), 1167–1174. 10.1097/JTO.0b013e3181e2f4f5 20592629

[B86] Xu D. Rowland S. E. Clark P. Giroux A. Cote B. Guiral S. (2008). MF63 [2-(6-chloro-1H-phenanthro[9,10-d]imidazol-2-yl)-isophthalonitrile], a selective microsomal prostaglandin E synthase-1 inhibitor, relieves pyresis and pain in preclinical models of inflammation. J. Pharmacol. Exp. Ther. 326 (3), 754–763. 10.1124/jpet.108.138776 18524979

[B87] Yoshimatsu K. Altorki N. K. Golijanin D. Zhang F. Jakobsson P. J. Dannenberg A. J. (2001). Inducible prostaglandin E synthase is overexpressed in non-small cell lung cancer. Clin. Cancer Res. 7 (9), 2669–2674. 11555578

[B88] Young M. M. Kester M. Wang H. G. (2013). Sphingolipids: regulators of crosstalk between apoptosis and autophagy. J. Lipid. Res. 54 (1), 5–19. 10.1194/jlr.R031278 23152582 PMC3520539

[B89] Zelenay S. van der Veen A. G. Bottcher J. P. Snelgrove K. J. Rogers N. Acton S. E. (2015). Cyclooxygenase-dependent tumor growth through evasion of immunity. Cell 162 (6), 1257–1270. 10.1016/j.cell.2015.08.015 26343581 PMC4597191

