# Supplementary material for: Inhibition of mPGES-1 or COX-2 Results in Different Proteomic and Lipidomic Profiles in A549 Lung Cancer Cells
Source: Front Pharmacol. 2019 Jun 7;10:636. doi: 10.3389/fphar.2019.00636 (PMC6567928; doi:10.3389/fphar.2019.00636)
Supplement: Supplementary file 1 [file DataSheet_1.pdf]

Supplementary Material

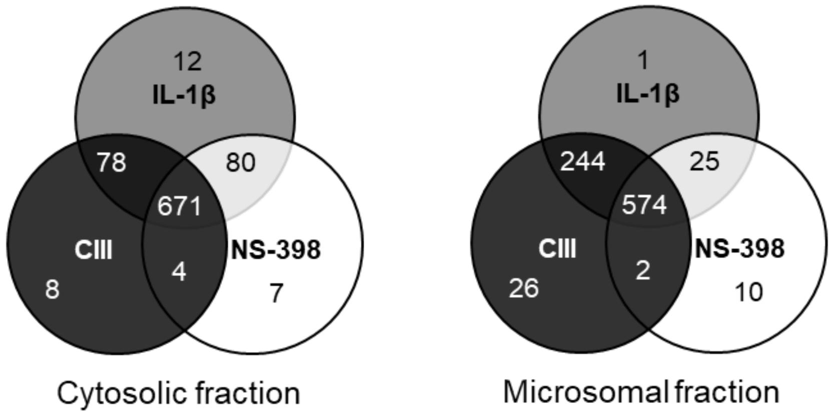

Supplementary Figure S1. Venn diagrams showing numbers of shared and unique protein identities across treatment groups (IL-1β, CIII, and NS-398) in the cytosolic and microsomal fractions.

**Top-30 proteins altered by IL-1β**				
UniProt ID	Protein name	Fraction	Fold-change to Ctrl	p-value
P36405	ADP-ribosylation factor-like protein 3	Cytosolic	1.45	7.6E-06*
Q92973	Transportin-1	Cytosolic	2.11	1.8E-05*
Q9P287	BRCA2 and CDKN1A-interacting protein	Cytosolic	0.04	2.8E-05*
Q15293	Reticulocalbin-1	Cytosolic	1.54	4.8E-05*
Q9NS56	E3 ubiquitin-protein ligase Topors	Cytosolic	1.61	5.0E-05*
Q9NZJ4	Sacsin	Microsomal	1.21	6.3E-05*
P17676	CCAAT/enhancer-binding protein beta	Cytosolic	1.51	6.4E-05*
P68036	Ubiquitin-conjugating enzyme E2 L3	Cytosolic	1.43	0.00012*
P46940	Ras GTPase-activating-like protein IQGAP1	Cytosolic	1.26	0.00013*
O95373	Importin-7	Cytosolic	1.65	0.00016*
Q9NR31	GTP-binding protein SAR1a	Cytosolic	2.01	0.00016*
Q9ULW5	Ras-related protein Rab-26	Cytosolic	1.86	0.00024*
Q01518	Adenylyl cyclase-associated protein 1	Cytosolic	1.96	0.00025*
Q86UK5	Limbin	Cytosolic	1.40	0.00025*
O60602	Toll-like receptor 5	Cytosolic	1.82	0.00029*
P37173	TGF-beta receptor type-2	Cytosolic	0.08	0.00031*
Q9UID3	Vacuolar protein sorting-associated protein 51 homolog	Cytosolic	0.33	0.00041*
Q9BX82	Zinc finger protein 471	Cytosolic	1.63	0.00043*
Q9H7M9	V-type immunoglobulin domain-containing suppressor of T-cell activation	Microsomal	0.72	0.00046*
A4D1S5	Ras-related protein Rab-19	Cytosolic	1.61	0.00051*
Q9Y2I1	Nischarin	Cytosolic	1.65	0.00051*
Q9H4M9	EH domain-containing protein 1	Cytosolic	1.99	0.00059*
P15121	Aldose reductase	Microsomal	0.09	0.00060*
Q04637	Eukaryotic translation initiation factor 4 gamma 1	Microsomal	0.09	0.00065*
O95147	Dual specificity protein phosphatase 14	Cytosolic	1.75	0.00068*
Q8WZ82	Esterase OVCA2	Cytosolic	1.28	0.00071*
P24534	Elongation factor 1-beta	Cytosolic	1.43	0.00077*
P26447	Protein S100-A4	Cytosolic	1.89	0.00094
Q92541	RNA polymerase-associated protein RTF1 homolog	Cytosolic	1.70	0.00097
P23528	Cofilin-1	Microsomal	0.19	0.0011

Supplementary Table S1. The list shows the top-30 proteins altered by IL-1β based on p-value. Statistical significance was tested using individual t-test (n=3) followed by Benjamini-Hochberg procedure (α=0.05). An asterisk (*) denotes significant p-value after Benjamini-Hochberg procedure.

Fatty acid	Ctrl	IL-1β	CIII	NS-398
C14:0	2.8 ± 1.0	2.1 ± 0.2	2.1 ± 0.1	2.2 ± 0.1
C16:0	23.4 ± 1.9	23.5 ± 1.3	23.4 ± 0.2	24.0 ± 1.2
C16:1	10.3 ± 0.6	10.9 ± 0.5	11.1 ± 0.4	10.2 ± 0.1
C18:0	11.3 ± 0.5	10.8 ± 0.6	10.3 ± 0.3	10.5 ± 0.2
C18:1	44.2 ± 1.5	44.5 ± 0.6	44.9 ± 0.4	44.6 ± 1.2
C18:2	0.8 ± 0.3	1.1 ± 0.03	1.0 ± 0.1	1.1 ± 0.1
C20:4(n-6)	4.3 ± 0.3	4.4 ± 0.3	4.0 ± 0.1	4.3 ± 0.1
C22:6(n-3)	2.8 ± 0.5	2.6 ± 0.4	3.2 ± 0.3	3.1 ± 0.3
SFA	37.5 ± 2.0	36.4 ± 0.7	35.8 ± 0.4	36.7 ± 1.1
MUFA	54.5 ± 1.0	55.4 ± 0.3	56.0 ± 0.3	54.8 ± 1.1
PUFA	8.0 ± 1.0	8.2 ± 0.7	8.2 ± 0.3	8.5 ± 0.4

Supplementary Table S2. Fatty acids were measured with GC-FID and expressed as mol% (mean ± SD, n=3) for one experiment. Statistical significance was tested using one-way ANOVA followed by individual t-test with Bonferroni correction ($p<0.05$). There was no statistical significant difference between the groups.

Phospholipid	Ctrl	IL-1β	CIII	NS-398
**LPC(16:1)**	10.63 ± 0.46	**11.81 ± 0.36**	**10.54 ± 0.09***	12.21 ± 0.96
**LPC(16:0)**	43.11 ± 3.81	**46.34 ± 1.55**	**45.47 ± 0.54**	**30.69 ± 0.92*#**
**LPC(18:2)**	3.09 ± 0.16	**3.02 ± 0.14**	**3.54 ± 0.19**	**2.52 ± 0.13*#**
**LPC(18:1)**	39.9 ± 3.13	**35.99 ± 0.96**	**37.09 ± 0.75**	**52.03 ± 0.47*#**
**LPC(20:4)**	1.25 ± 0.18	**1.03 ± 0.05**	**1.29 ± 0.06***	**1.03 ± 0.02#**
**LPC(22:6)**	2.03 ± 0.21	1.8 ± 0.19	**2.07 ± 0.18**	**1.52 ± 0.05#**
PC(32:4)	0.02 ± 0.002	0.02 ± 0.004	0.02 ± 0.004	0.02 ± 0.001
PC(32:3)	0.34 ± 0.04	0.44 ± 0.09	0.47 ± 0.08	0.39 ± 0.01
PC(32:2)	6.31 ± 0.96	6.97 ± 0.77	7.41 ± 0.91	5.95 ± 0.03
PC(32:1)	22.57 ± 1.18	21.52 ± 1.15	20.45 ± 0.11	21.57 ± 0.7
**PC(32:0)**	**6.13 ± 0.1***	**6.94 ± 0.28**	7.3 ± 0.15	7.58 ± 0.41
PC(34:5)	0.03 ± 0.004	0.03 ± 0.01	0.03 ± 0.004	0.03 ± 0.001
PC(34:4)	0.22 ± 0.02	0.23 ± 0.01	0.24 ± 0.02	0.21 ± 0.01
PC(34:3)	1.47 ± 0.15	1.32 ± 0.13	1.6 ± 0.12	1.38 ± 0.09
**PC(34:2)**	12.87 ± 0.88	**11.31 ± 0.32**	**12.71 ± 0.4***	11.87 ± 0.18
PC(34:1)	24.8 ± 1.26	24.5 ± 1.11	23.74 ± 1.82	24.53 ± 0.95
**PC(34:0)**	3.14 ± 0.3	**3.31 ± 0.13**	**2.89 ± 0.05***	**3.33 ± 0.06#**
PC(36:6)	0.07 ± 0.01	0.08 ± 0.01	0.07 ± 0.002	0.07 ± 0.01
PC(36:5)	0.27 ± 0.03	0.28 ± 0.02	0.24 ± 0.02	0.27 ± 0.01
PC(36:4)	1.08 ± 0.11	0.96 ± 0.02	0.98 ± 0.01	0.98 ± 0.04
PC(36:3)	2.1 ± 0.14	2.03 ± 0.16	2.25 ± 0.07	2.06 ± 0.08
PC(36:2)	10.47 ± 0.14	10.85 ± 1.24	10.82 ± 0.32	10.63 ± 0.19
PC(36:1)	5.28 ± 0.95	6.01 ± 0.48	5.6 ± 0.35	5.98 ± 0.29
PC(36:0)	0.77 ± 0.14	0.89 ± 0.07	0.77 ± 0.06	0.81 ± 0.04
PC(38:7)	0.1 ± 0.01	0.14 ± 0.01	0.12 ± 0.01	0.14 ± 0.01
**PC(38:6)**	0.45 ± 0.05	**0.5 ± 0.01**	**0.42 ± 0.004***	**0.45 ± 0.004*#**
PC(38:5)	0.44 ± 0.02	0.46 ± 0.02	0.49 ± 0.02	0.49 ± 0.01
**PC(38:4)**	0.69 ± 0.04	**0.76 ± 0.04**	**0.9 ± 0.01***	**0.78 ± 0.02#**
PC(40:7)	0.17 ± 0.01	0.21 ± 0.01	0.2 ± 0.01	0.21 ± 0.02
PC(40:6)	0.2 ± 0.03	0.26 ± 0.01	0.29 ± 0.03	0.26 ± 0
PE(30:0)	0.31 ± 0.06	0.45 ± 0.09	0.42 ± 0.03	0.37 ± 0.04
PE(32:2)	1.56 ± 0.13	2.15 ± 0.32	1.93 ± 0.06	1.88 ± 0.14
PE(32:1)	6.65 ± 0.29	7.65 ± 0.61	8.81 ± 0.63	7.65 ± 0.56
PE(32:0)	1.52 ± 0.12	1.77 ± 0.28	1.98 ± 0.11	1.67 ± 0.16

PE(34:5)	1.51 ± 0.11	1.77 ± 0.29	1.98 ± 0.11	1.66 ± 0.16
PE(34:4)	0.21 ± 0.003	0.21 ± 0.02	0.21 ± 0.01	0.21 ± 0.02
PE(34:3)	0.86 ± 0.02	0.92 ± 0.02	0.94 ± 0.06	0.93 ± 0.04
PE(34:2)	13.47 ± 0.64	14.75 ± 2.22	14.33 ± 0.95	14.49 ± 1.15
PE(34:1)	24.09 ± 0.73	24.98 ± 1.06	24.84 ± 1.19	23.41 ± 0.45
PE(34:0)	2.37 ± 0.09	2.45 ± 0.15	2.5 ± 0.15	2.32 ± 0.09
PE(36:6)	0.12 ± 0.02	0.13 ± 0.02	0.12 ± 0.01	0.11 ± 0.01
PE(36:5)	0.86 ± 0.07	0.91 ± 0.12	0.73 ± 0.02	0.85 ± 0.03
PE(36:4)	5.59 ± 0.31	5.43 ± 0.36	5.01 ± 0.03	5.00 ± 0.20
PE(36:3)	4.51 ± 0.24	4.18 ± 0.27	4.01 ± 0.04	4.03 ± 0.08
PE(36:2)	15.98 ± 0.88	14.18 ± 1.5	14.07 ± 1.64	16.2 ± 1.01
PE(36:1)	9.92 ± 0.29	8.43 ± 0.97	8.95 ± 0.61	9.38 ± 0.54
PE(36:0)	2.42 ± 0.01	2.19 ± 0.19	2.22 ± 0.1	2.33 ± 0.07
PE(38:7)	0.17 ± 0.03	0.2 ± 0.01	0.18 ± 0.01	0.2 ± 0.02
PE(38:6)	1.77 ± 0.12	1.89 ± 0.03	1.81 ± 0.06	1.76 ± 0.04
**PE(38:5)**	**5.16 ± 0.08***	**4.59 ± 0.04**	**4.13 ± 0.11***	4.62 ± 0.2
PE(40:8)	0.05 ± 0.01	0.04 ± 0.01	0.07 ± 0.01	0.06 ± 0.01
PE(40:6)	0.9 ± 0.004	0.73 ± 0.09	0.77 ± 0.12	0.86 ± 0.03
PS(34:2)	3.82 ± 0.1	4.26 ± 0.2	4.48 ± 0.14	3.59 ± 0.37
PS(34:1)	32.17 ± 1.92	37.09 ± 1.95	36.11 ± 1.9	34.63 ± 2.2
PS(34:0)	3.36 ± 0.04	3.69 ± 0.35	3.89 ± 0.21	3.54 ± 0.2
PS(36:3)	1.61 ± 0.11	1.57 ± 0.23	1.72 ± 0.03	1.58 ± 0.05
PS(36:2)	16.62 ± 0.44	16.91 ± 1.16	16.57 ± 1.22	16.66 ± 0.43
PS(36:1)	36.94 ± 1.28	31.68 ± 2.67	32.66 ± 2.57	34.79 ± 2.9
PS(36:0)	4.29 ± 0.56	3.84 ± 0.38	3.88 ± 0.43	4.29 ± 0.13
**PS(40:6)**	1.19 ± 0.11	0.95 ± 0.2	**0.68 ± 0.02**	**0.91 ± 0.06#**

Supplementary Table S3. Phospholipids were measured using LC-MS/MS and expressed as area% (mean ± SD, n=3) for one experiment. Statistical significance was tested using one-way ANOVA followed by individual t-test with Bonferroni correction ($p<0.05$). Phospholipids altered by treatment are highlighted in bold. Asterisk (*) denotes significant difference to IL-1β and hashtag (#) denotes significant difference between CIII and NS-398.

Sphingolipid	Ctrl	IL-1β	CIII	NS-398
**Cer C14:0**	0.45 ± 0.05	**0.43 ± 0.04**	**0.44 ± 0.05**	**0.23 ± 0.04*#**
**Cer C16:0**	10.91 ± 1.7	**9.97 ± 0.45**	8.19 ± 0.81	**5.97 ± 1.48***
Cer C18:0	0.39 ± 0.06	0.49 ± 0.07	0.45 ± 0.01	0.36 ± 0.1
**Cer C20:0**	0.15 ± 0.03	**0.18 ± 0.01**	0.18 ± 0.03	**0.11 ± 0.01***
**Cer C22:0**	1.38 ± 0.24	**1.33 ± 0.04**	**1.48 ± 0.22**	**0.7 ± 0.17*#**
**Cer C24:1**	7.99 ± 1.4	**6.77 ± 0.43**	**7.05 ± 0.78**	**3.22 ± 0.85*#**
**Cer C24:0**	8.53 ± 1.94	**8.01 ± 0.28**	6.83 ± 1.27	**3.37 ± 0.87***
SM C16:0	504 ± 68	469 ± 45	421 ± 67	314 ± 95
SM C18:1	2.77 ± 0.19	2.57 ± 0.25	3.55 ± 0.98	1.61 ± 0.67
SM C18:0	10.91 ± 1.74	11.43 ± 1.0	12.54 ± 1.09	7.93 ± 2.4
SM C24:1	229 ± 32	206 ± 18	231 ± 35	131 ± 42
SM C24:0	106.1 ± 13.1	98.6 ± 10.0	99.85 ± 16.4	54.6 ± 17.8
**HexCer C16:0**	19.85 ± 2.22	**25.7 ± 3.95**	**14.68 ± 1.59***	12.55 ± 4.74
HexCer C18:1	0.19 ± 0.06	0.26 ± 0.1	0.16 ± 0.05	0.18 ± 0.03
HexCer C18:0	1.27 ± 0.23	1.55 ± 0.18	1.09 ± 0.13	0.78 ± 0.27
HexCer C24:1	26.84 ± 3.19	30.31 ± 3.67	23.86 ± 4.28	15.4 ± 5.3
**LacCer C16:0**	1.77 ± 0.48	**1.94 ± 0.38**	**1.81 ± 0.21**	**0.79 ± 0.24*#**
LacCer C24:1	1.59 ± 0.39	1.39 ± 0.19	1.29 ± 0.25	0.93 ± 0.22
**DhCer C16:0**	1.95 ± 0.41	**1.74 ± 0.43**	**7.89 ± 0.53***	**1.54 ± 0.44#**
Sphingosine	38.06 ± 14.14	60.8 ± 8.8	45.56 ± 10.36	37.38 ± 11.72
**Sphinganine**	11.75 ± 1.01	**15.6 ± 2.7**	29.23 ± 5.25	**10.69 ± 1.76#**

Supplementary Table S4. Sphingolipids were measured with LC-MS/MS and expressed as pmol per million cells (mean ± SD, n=3) of one experiment. Statistical significance was tested using one-way ANOVA followed by individual t-test with Bonferroni correction ($p<0.05$). Sphingolipids altered by treatment are highlighted in bold. Asterisk (*) denotes significant difference to IL-1β and hashtag (#) denotes significant difference between CIII and NS-398.

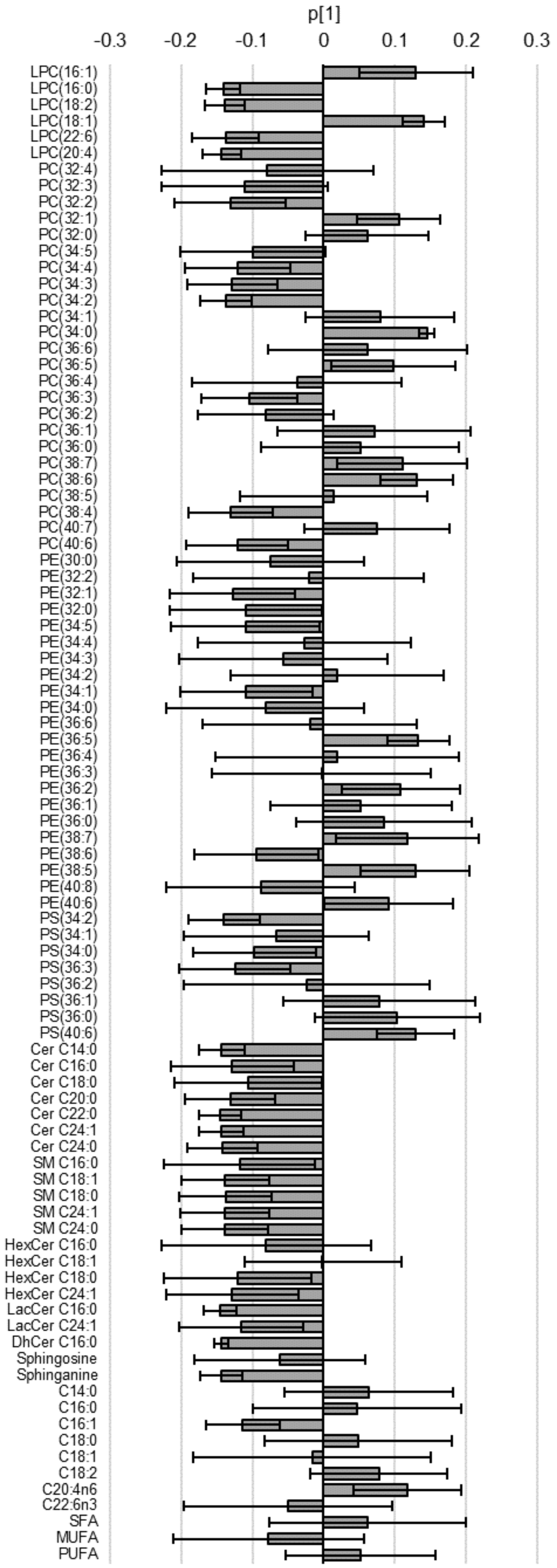
p[1]
-0.3
-0.2
-0.1
0
0.1
0.2
0.3
LPC(16:1)
LPC(16:0)
LPC(18:2)
LPC(18:1)
LPC(22:6)
LPC(20:4)
PC(32:4)
PC(32:3)
PC(32:2)
PC(32:1)
PC(32:0)
PC(34:5)
PC(34:4)
PC(34:3)
PC(34:2)
PC(34:1)
PC(34:0)
PC(36:6)
PC(36:5)
PC(36:4)
PC(36:3)
PC(36:2)
PC(36:1)
PC(36:0)
PC(38:7)
PC(38:6)
PC(38:5)
PC(38:4)
PC(40:7)
PC(40:6)
PE(30:0)
PE(32:2)
PE(32:1)
PE(32:0)
PE(34:5)
PE(34:4)
PE(34:3)
PE(34:2)
PE(34:1)
PE(34:0)
PE(36:6)
PE(36:5)
PE(36:4)
PE(36:3)
PE(36:2)
PE(36:1)
PE(36:0)
PE(38:7)
PE(38:6)
PE(38:5)
PE(40:8)
PE(40:6)
PS(34:2)
PS(34:1)
PS(34:0)
PS(36:3)
PS(36:2)
PS(36:1)
PS(36:0)
PS(40:6)
Cer C14:0
Cer C16:0
Cer C18:0
Cer C20:0
Cer C22:0
Cer C24:1
Cer C24:0
SM C16:0
SM C18:1
SM C18:0
SM C24:1
SM C24:0
HexCer C16:0
HexCer C18:1
HexCer C18:0
HexCer C24:1
LacCer C16:0
LacCer C24:1
DhCer C16:0
Sphingosine
Sphinganine
C14:0
C16:0
C16:1
C18:0
C18:1
C18:2
C20:4n6
C22:6n3
SFA
MUFA
PUFA

Supplementary Figure S2. Loadings obtained from PCA of lipidomics data for CIII and NS-398. A high absolute loading value indicated a lipid important for the separation between CIII and NS-398 in principle component 1. Scores plot is shown in Figure 3A.

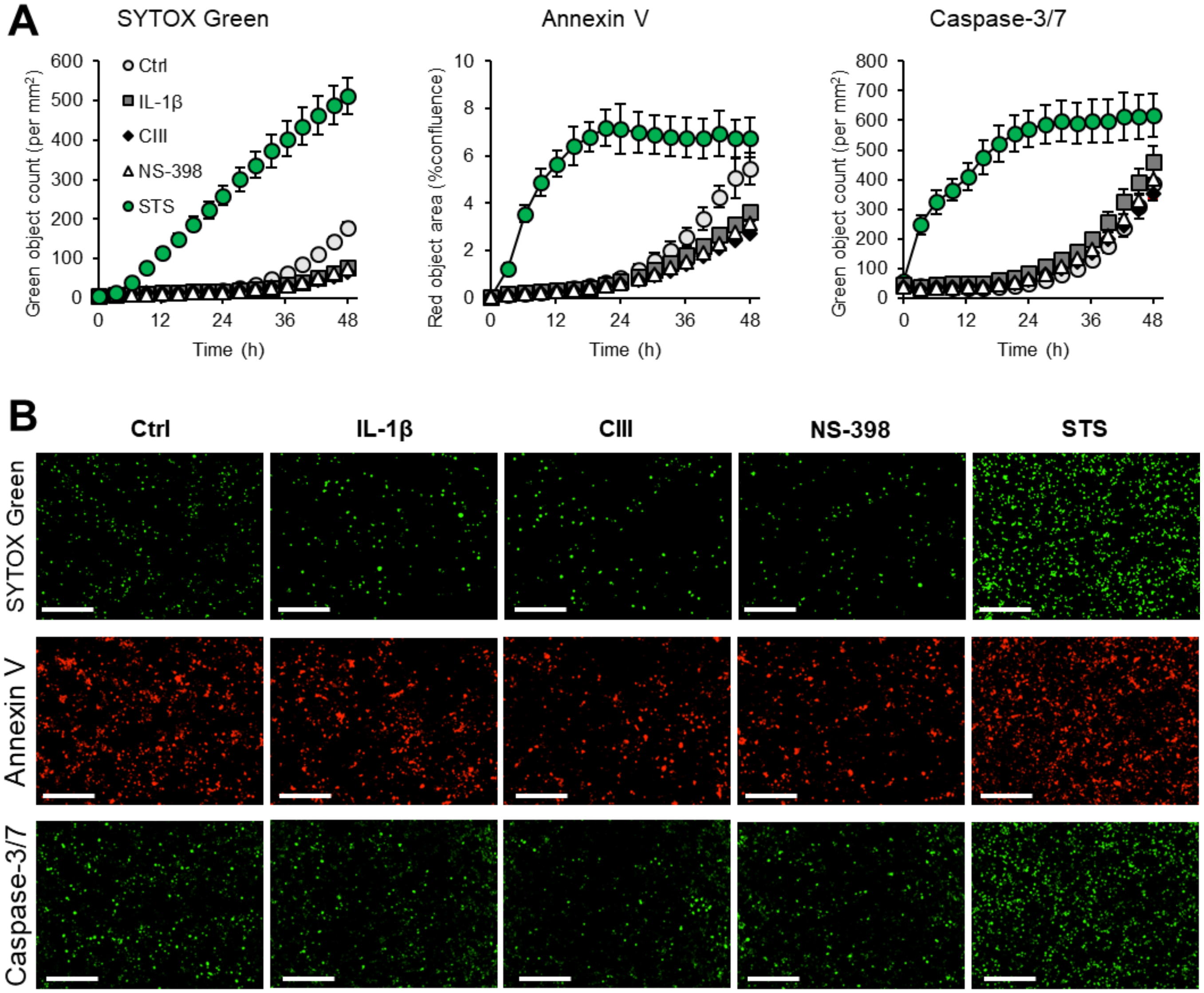

Supplementary Figure S3. Effect on cell viability and apoptosis by CIII and NS-398. **(A)** Quantification of cell viability using SYTOX Green and apoptosis using annexin V and caspase-3/7 reagents. Data are presented as mean ± SD (n=3) with Ctrl (light grey circle, ○), IL-1β (dark grey square, ■), CIII (black diamond, ♦), NS-398 (white triangle, Δ), and positive control staurosporine (STS, green circle, ●) for one experiment. **(B)** Representative pictures showing fluorescence at 48 hrs of SYTOX green (green), annexin V (red), and caspase-3/7 (green). There was no difference between the two inhibitors.

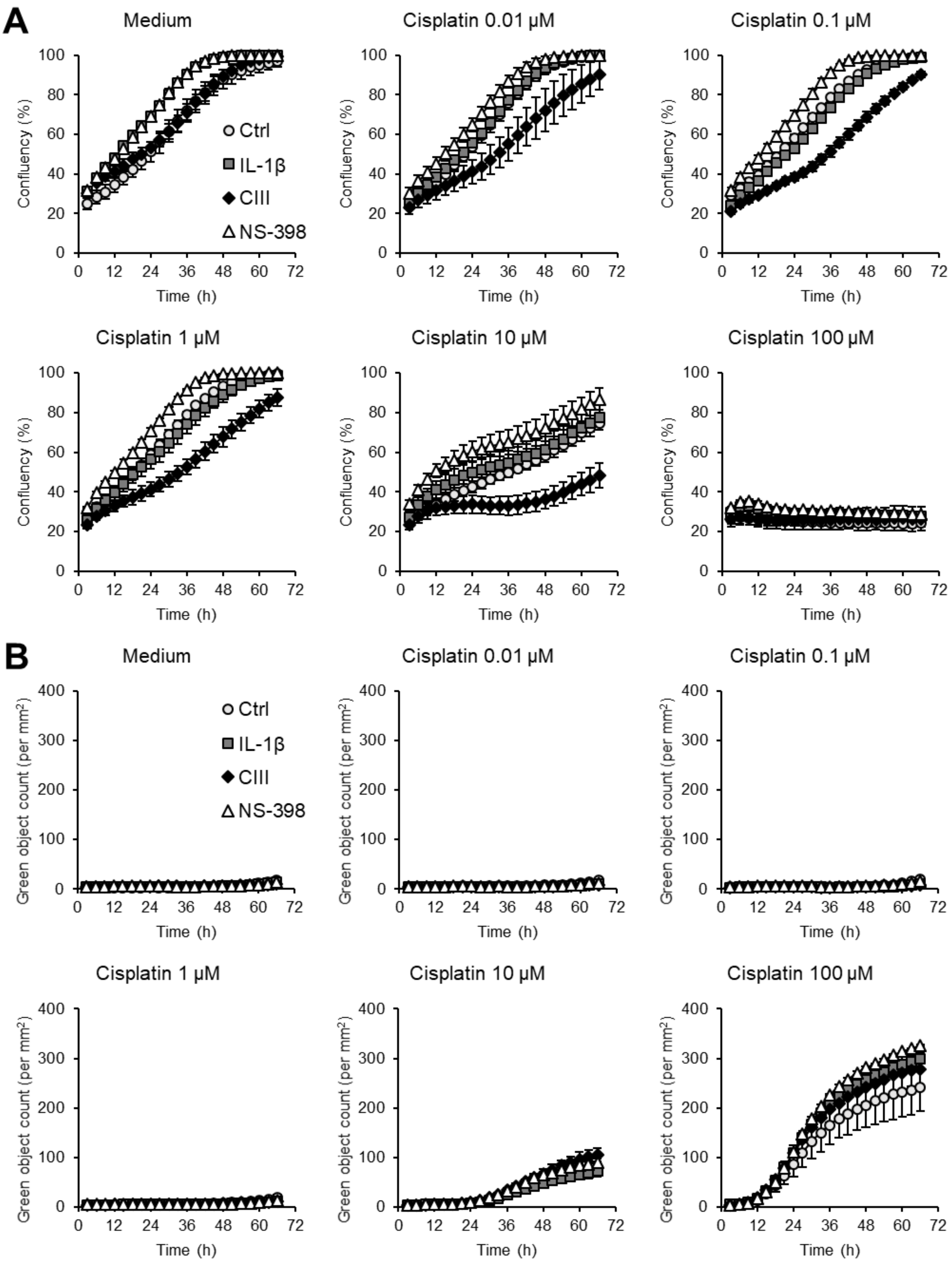
A
Medium
Cisplatin 0.01 µM
Cisplatin 0.1 µM
Cisplatin 1 µM
Cisplatin 10 µM
Cisplatin 100 µM
Confluency (%)
Time (h)
Ctrl
IL-1β
CIII
NS-398
B
Green object count (per mm²)

C

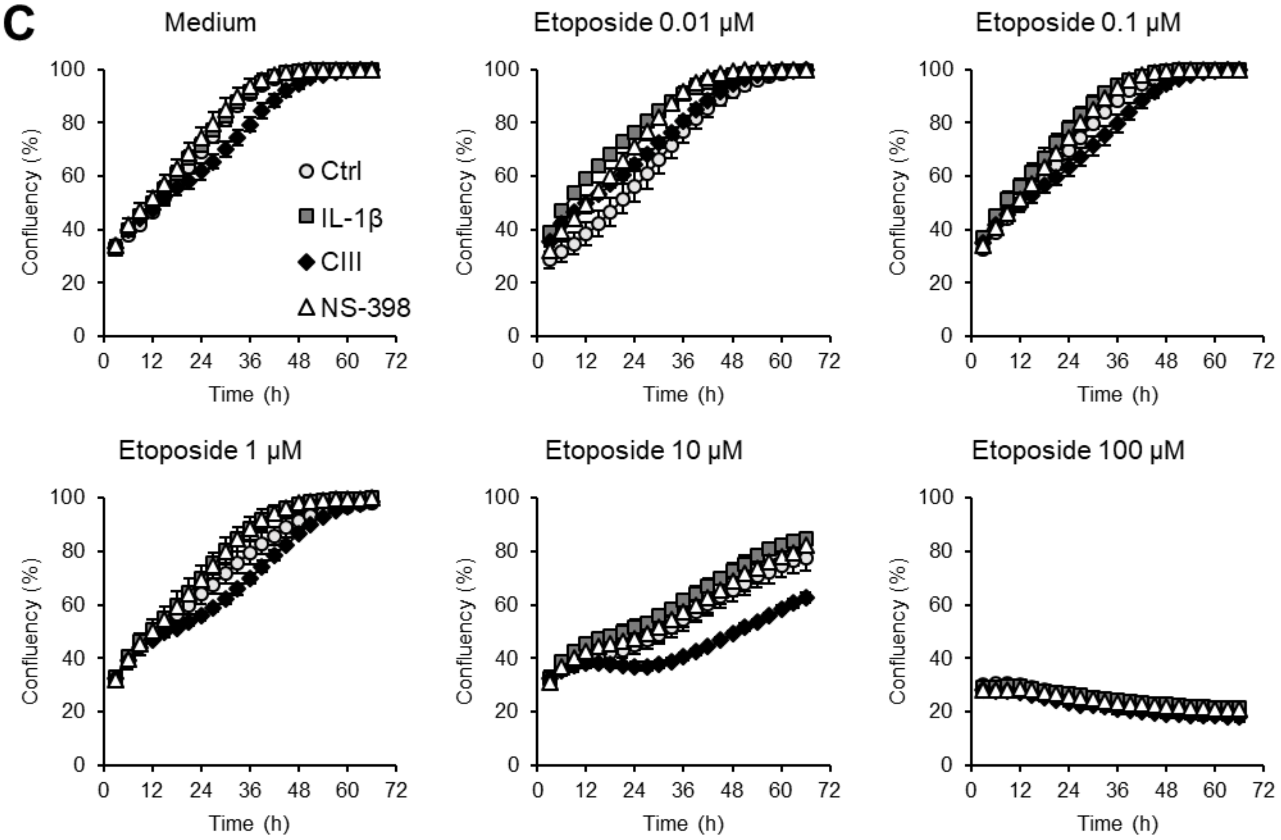
Medium
Etoposide 0.01 µM
Etoposide 0.1 µM
Etoposide 1 µM
Etoposide 10 µM
Etoposide 100 µM
Confluency (%)
Time (h)
Ctrl
IL-1β
CIII
NS-398

D

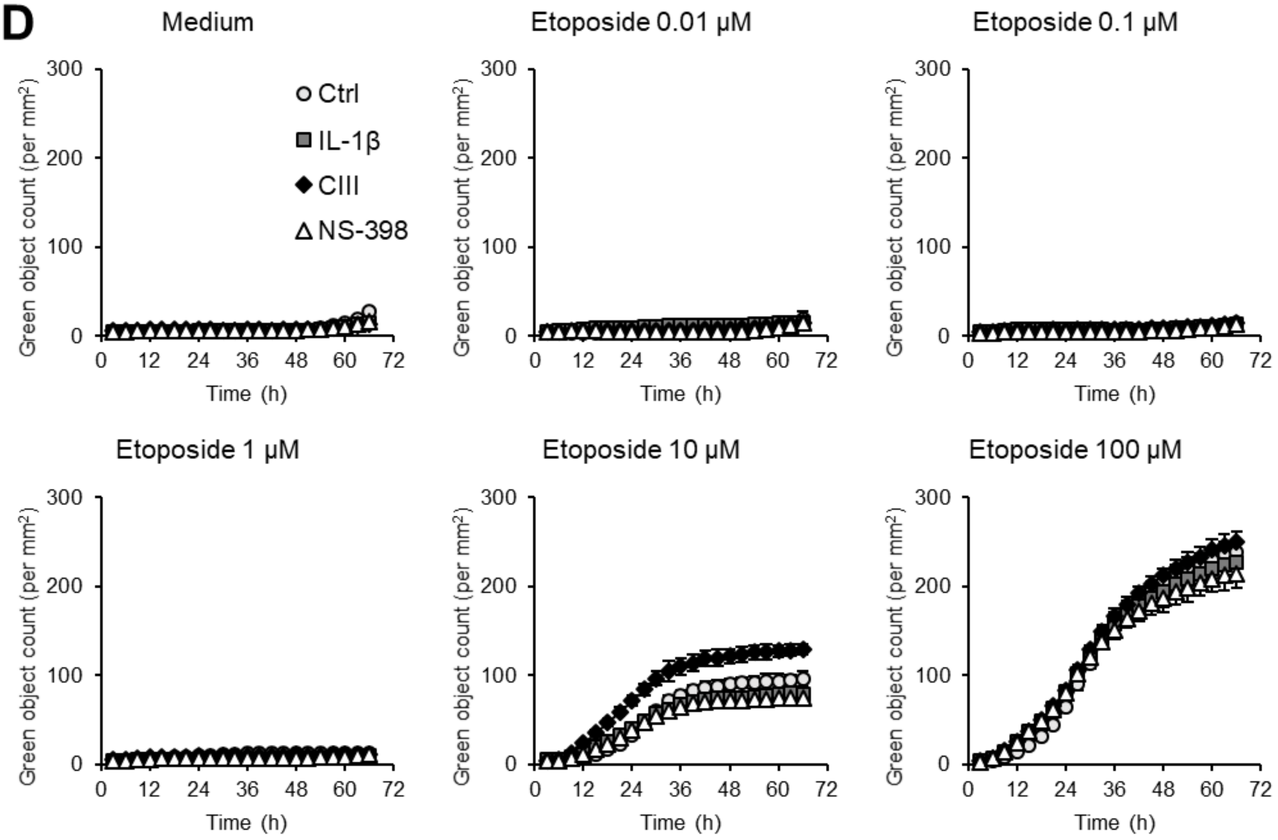
Medium
Etoposide 0.01 µM
Etoposide 0.1 µM
Etoposide 1 µM
Etoposide 10 µM
Etoposide 100 µM
Green object count (per mm²)
Time (h)
Ctrl
IL-1β
CIII
NS-398

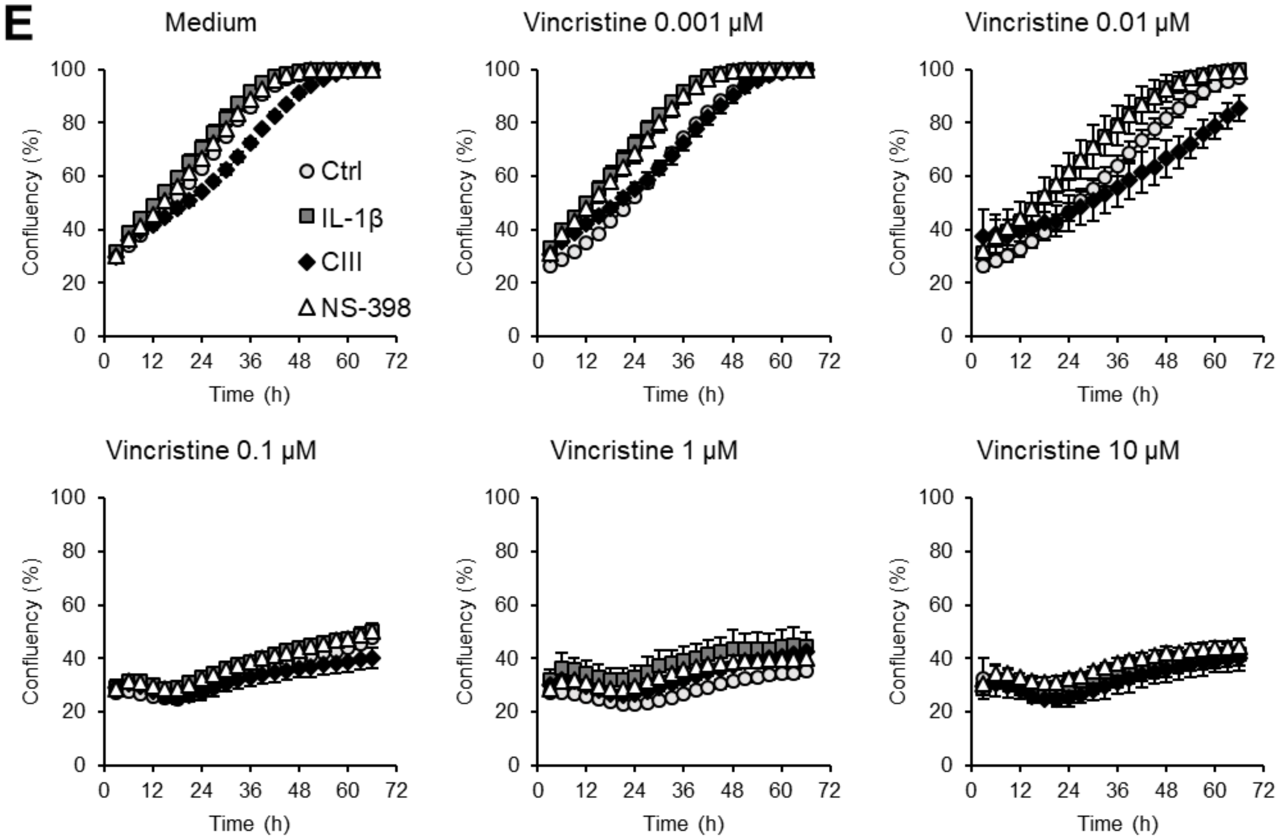
E
Medium
Vincristine 0.001 µM
Vincristine 0.01 µM
Vincristine 0.1 µM
Vincristine 1 µM
Vincristine 10 µM
Confluency (%)
Time (h)
Ctrl
IL-1β
CIII
NS-398

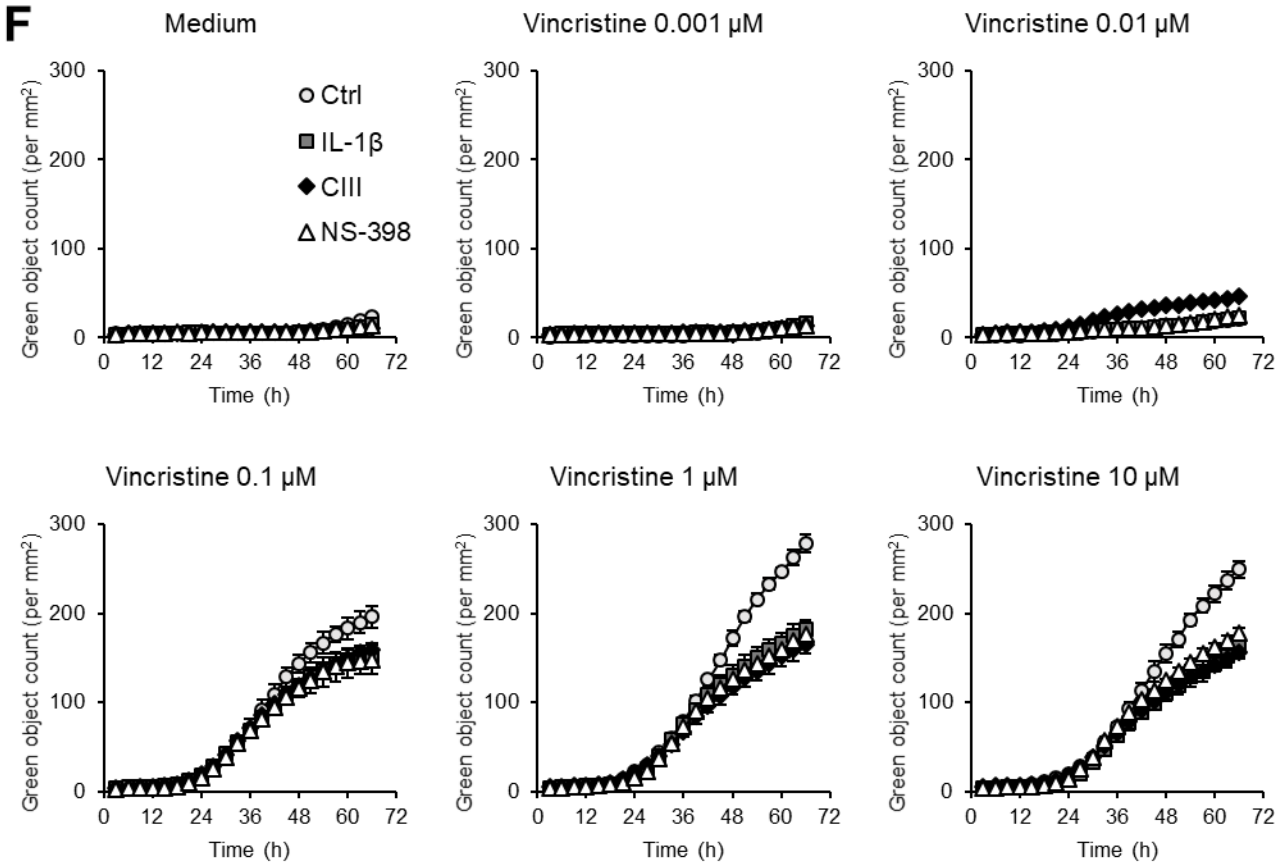
F
Medium
Vincristine 0.001 µM
Vincristine 0.01 µM
Vincristine 0.1 µM
Vincristine 1 µM
Vincristine 10 µM
Green object count (per mm²)
Time (h)
Ctrl
IL-1β
CIII
NS-398

Supplementary Figure S4. Effect on cell proliferation and cell death by CIII and NS-398 in combination with cytostatic drugs. Measurement of cell proliferation by confluency after co-treatment with cisplatin **(A)**, etoposide **(C)**, or vincristine **(E)**. Quantification of cell death by SYTOX Green after co-treatment with cisplatin **(B)**, etoposide **(D)**, or vincristine **(F)**. Data are presented as mean ± SD (n=3) with Ctrl (light grey circle, ○), IL-1β (dark grey square, ■), CIII (black diamond, ♦), and NS-398 (white triangle, Δ) from one experiment. The experiment was performed two times in triplicates. The second experiment is presented in Supplementary Figure S5.

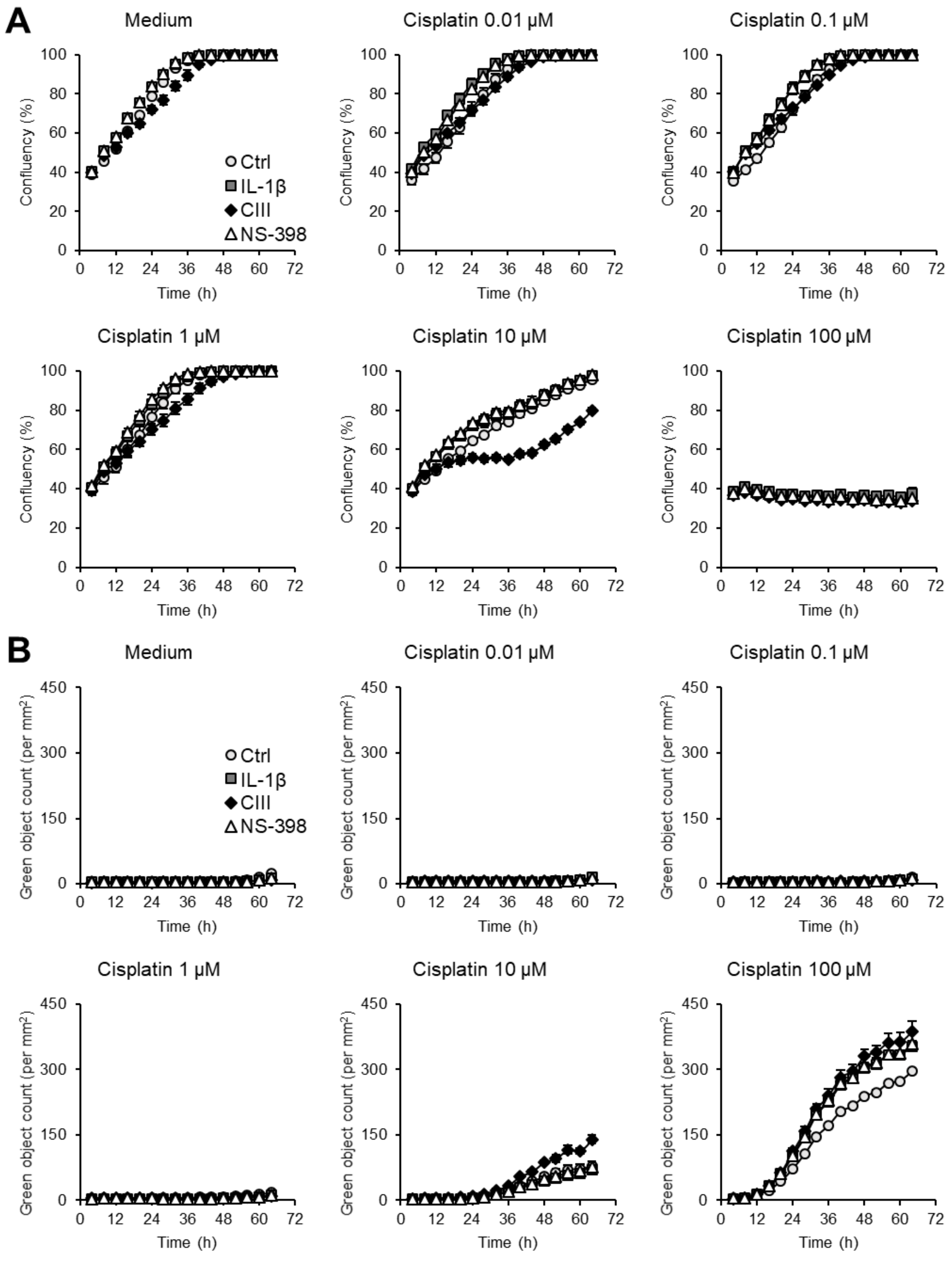
A
Medium
Cisplatin 0.01 µM
Cisplatin 0.1 µM
Cisplatin 1 µM
Cisplatin 10 µM
Cisplatin 100 µM
Confluency (%)
Time (h)
Ctrl
IL-1β
CIII
NS-398
B
Medium
Cisplatin 0.01 µM
Cisplatin 0.1 µM
Cisplatin 1 µM
Cisplatin 10 µM
Cisplatin 100 µM
Green object count (per mm2)
Time (h)
Ctrl
IL-1β
CIII
NS-398

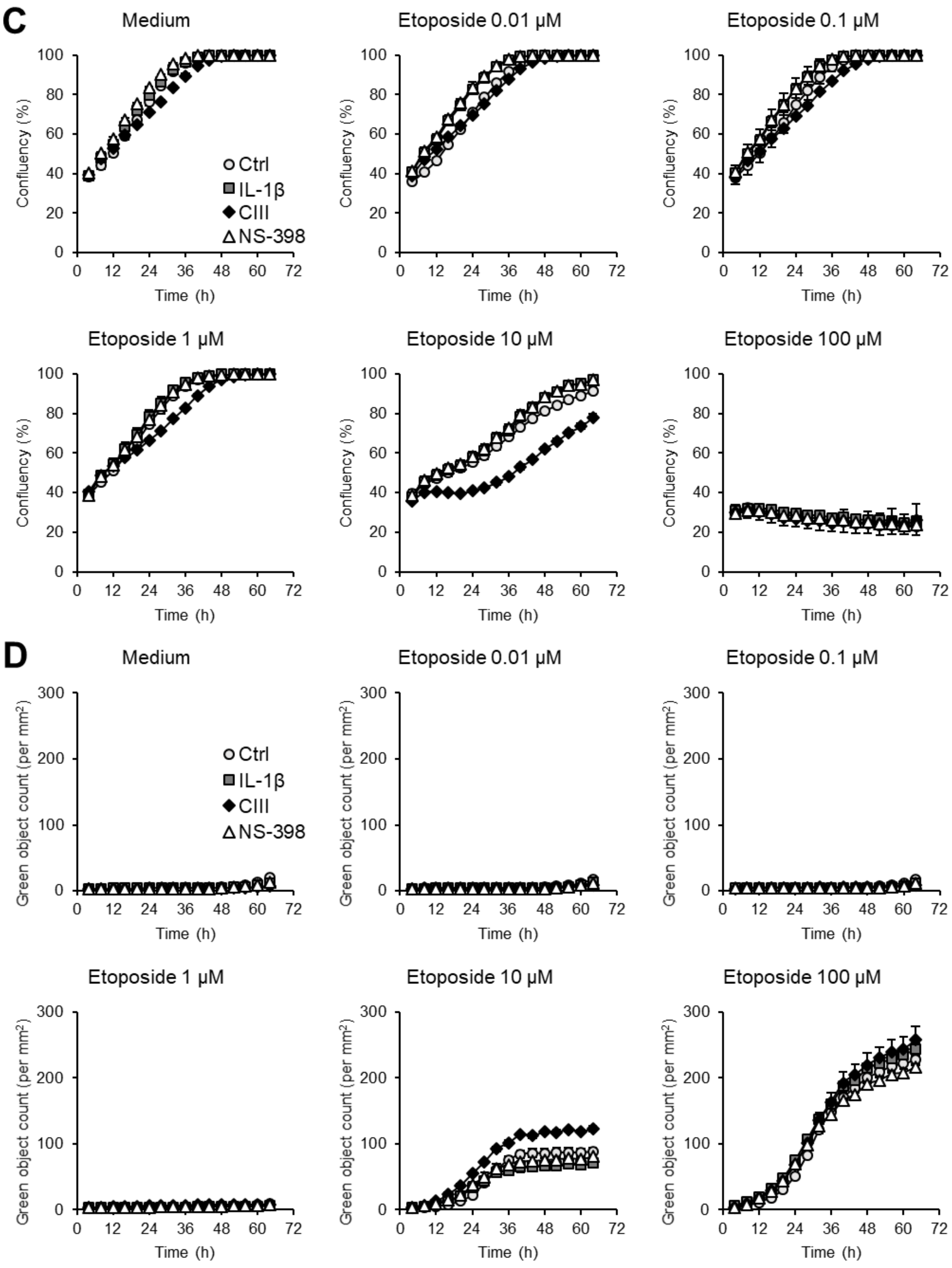
C
Medium
Etoposide 0.01 µM
Etoposide 0.1 µM
Etoposide 1 µM
Etoposide 10 µM
Etoposide 100 µM
Confluency (%)
Time (h)
Ctrl
IL-1β
CIII
NS-398
D
Medium
Etoposide 0.01 µM
Etoposide 0.1 µM
Etoposide 1 µM
Etoposide 10 µM
Etoposide 100 µM
Green object count (per mm^2)
Time (h)
Ctrl
IL-1β
CIII
NS-398

E

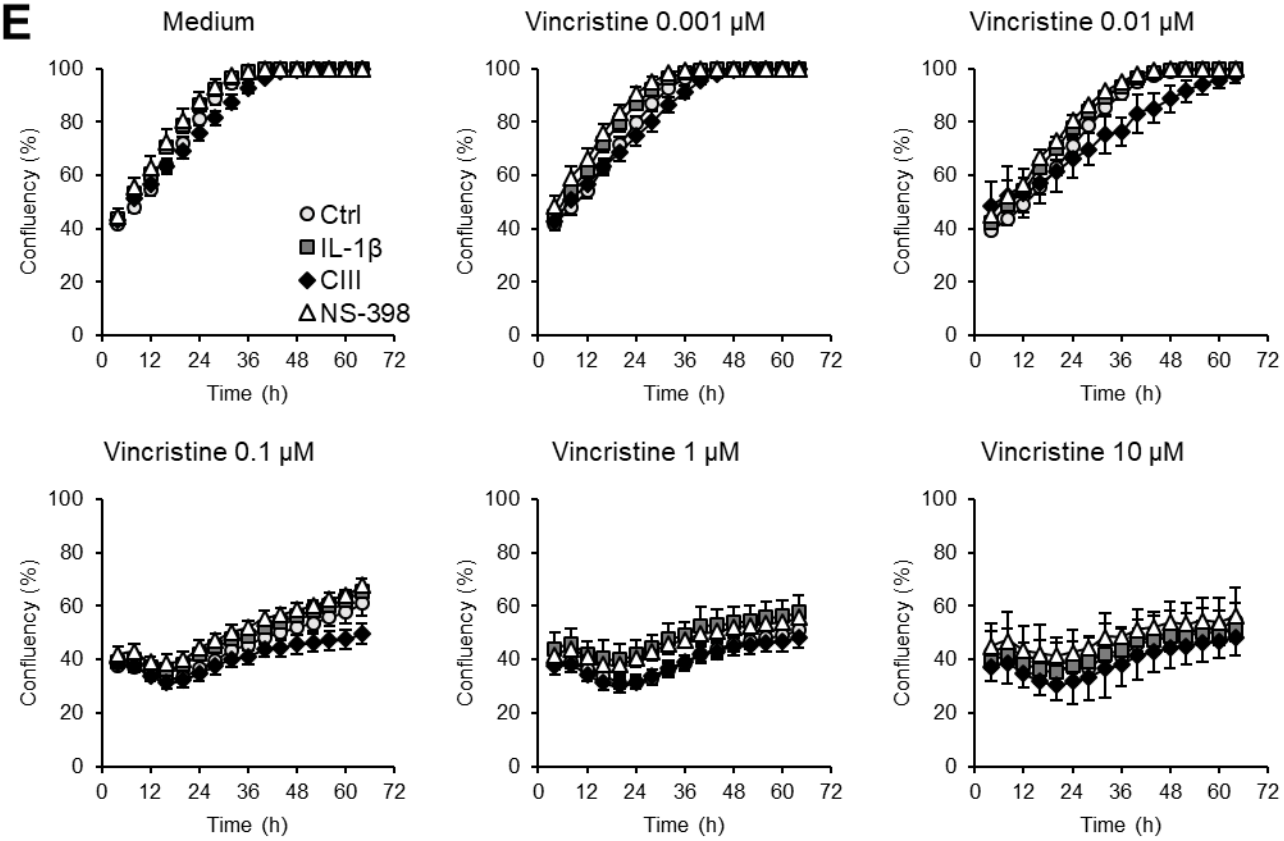
Medium
Vincristine 0.001 μM
Vincristine 0.01 μM
Vincristine 0.1 μM
Vincristine 1 μM
Vincristine 10 μM
Confluency (%)
Time (h)
0
20
40
60
80
100
0
12
24
36
48
60
72
Ctrl
IL-1β
CIII
NS-398

F

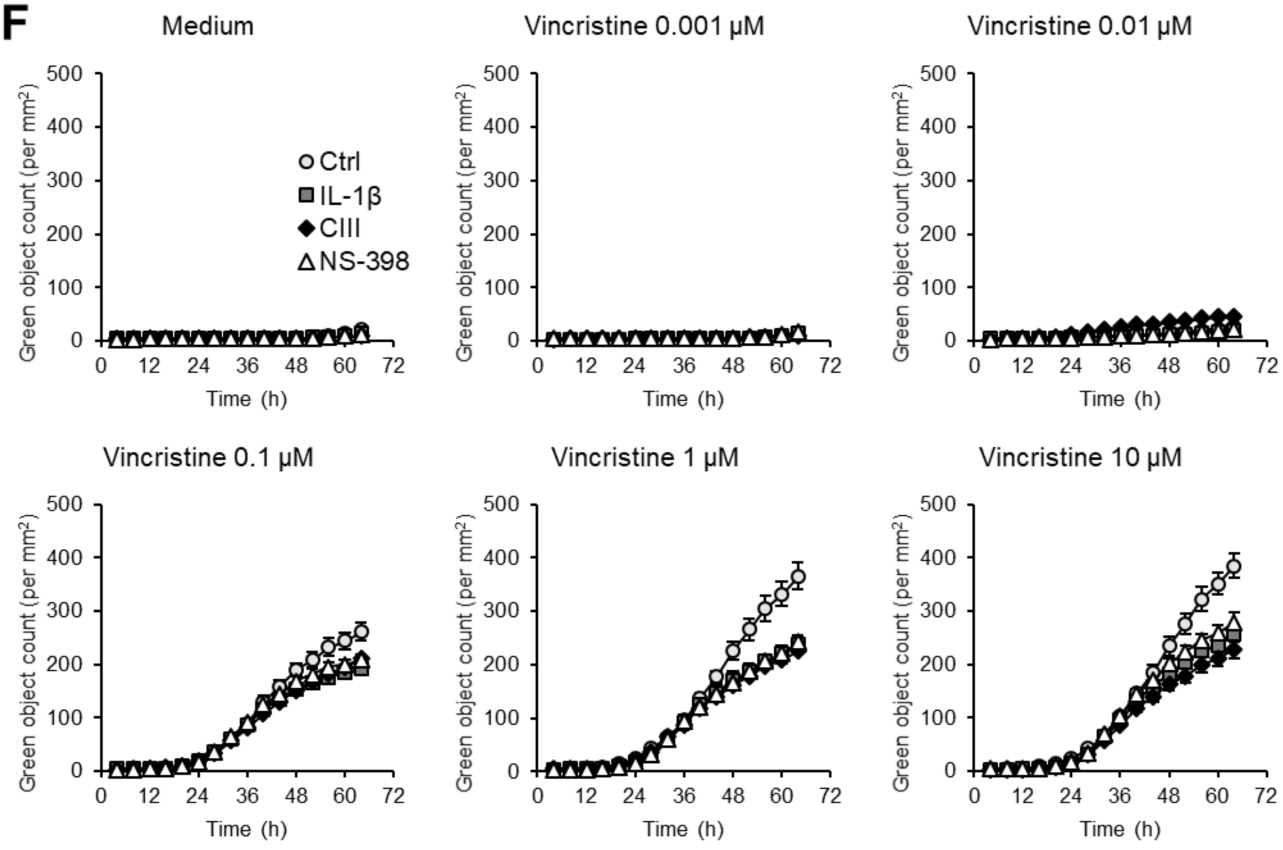
Medium
Vincristine 0.001 μM
Vincristine 0.01 μM
Vincristine 0.1 μM
Vincristine 1 μM
Vincristine 10 μM
Green object count (per mm^2)
Time (h)
0
100
200
300
400
500
0
12
24
36
48
60
72
Ctrl
IL-1β
CIII
NS-398

Supplementary Figure S5. Effect on cell proliferation and cell death by CIII and NS-398 in combination with cytostatic drugs. Measurement of cell proliferation by confluency after co-treatment with cisplatin **(A)**, etoposide **(C)**, or vincristine **(E)**. Quantification of cell death by SYTOX Green after co-treatment with cisplatin **(B)**, etoposide **(D)**, or vincristine **(F)**. Data are presented as mean ± SD (n=3) with Ctrl (light grey circle, ○), IL-1β (dark grey square, ■), CIII (black diamond, ♦), and NS-398 (white triangle, Δ) from one experiment. The experiment was performed two times in triplicates. The first experiment is presented in Supplementary Figure S4.
